# (Antibiotic-Resistant) *E. coli* in the Dutch–German Vecht Catchment—Monitoring and Modeling

**DOI:** 10.1021/acs.est.2c00218

**Published:** 2022-06-03

**Authors:** Eri van Heijnsbergen, Gunnar Niebaum, Volker Lämmchen, Alicia Borneman, Lucia Hernández Leal, Jörg Klasmeier, Heike Schmitt

**Affiliations:** † Wetsus, European Centre of Excellence for Sustainable Water Technology, Oostergoweg 9, 8911 MA Leeuwarden, The Netherlands; ‡ Institute of Environmental Systems Research, Osnabrück University, Barbarastraße 12, D-49076, Osnabrück, Germany; § Institute for Risk Assessment Sciences, Utrecht University, Yalelaan 2, 3584 CM Utrecht, The Netherlands

**Keywords:** *E. coli*, ESBL-producing *E. coli*, carbapenemase-producing *E. coli*, geo-referenced modeling, microbial water quality, aquatic exposure assessment

## Abstract

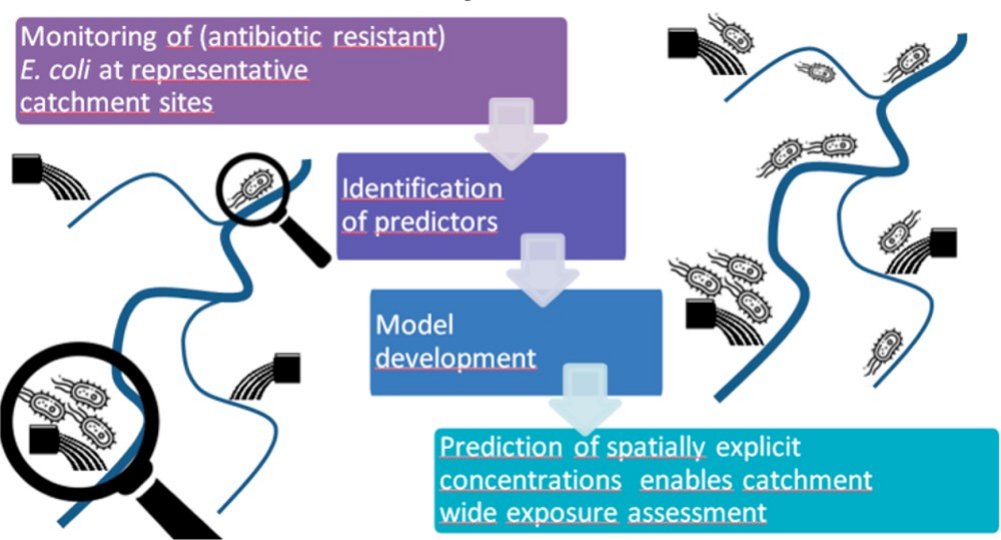

Fecally contaminated waters can be a source for human infections. We investigated the occurrence of fecal indicator bacteria (*E. coli*) and antibiotic-resistant *E. coli*, namely, extended spectrum beta-lactamase (ESBL)-producing *E. coli* (ESBL-EC) and carbapenemase-producing *E. coli* (CP-EC) in the Dutch–German transboundary catchment of the Vecht River. Over the course of one year, bacterial concentrations were monitored in wastewater treatment plant (WWTP) influents and effluents and in surface waters with and without WWTP influence. Subsequently, the GREAT-ER model was adopted for the prediction of (antibiotic-resistant) *E. coli* concentrations. The model was parametrized and evaluated for two distinct scenarios (average flow scenario, dry summer scenario). Statistical analysis of WWTP monitoring data revealed a significantly higher (factor 2) proportion of ESBL-EC among *E. coli* in German compared to Dutch WWTPs. CP-EC were present in 43% of influent samples. The modeling approach yielded spatially accurate descriptions of microbial concentrations for the average flow scenario. Predicted *E. coli* concentrations exceed the threshold value of the Bathing Water Directive for a good bathing water quality at less than 10% of potential swimming sites in both scenarios. During a single swimming event up to 61 CFU of ESBL-EC and less than 1 CFU of CP-EC could be taken up by ingestion.

## Abstract 2

This work establishes a combined approach of monitoring and modeling to predict spatially explicit in-stream concentrations of (antibiotic-resistant) *E. coli*.

## 1 Introduction

The application of antibiotics to treat previously incurable diseases has improved both life quality and expectancy. However, with the increasing use of antibiotics, the relative number of diseases caused by antibiotic-resistant pathogens has also increased in the past few decades.^1^ The emission of fecal bacteria into the environment from human excrements occurs via discharge of (treated) wastewater.^2,3^ This also encompasses many antibiotic-resistant species. Besides these point sources, diffuse sources are comprised of (i) direct runoff from areas with fecal contamination, e.g., from manure fertilization,^4^ (ii) direct drop-off of feces by pasture animals with direct water access,^5^ (iii) excretions of wildlife,^6^ especially water fowl,^7,8^ and (iv) remobilization of particle bound and trapped bacteria, from the sediments.^9^

Fecal contamination of surface water is usually assessed via indicator bacteria like the intestinal bacteria *Escherichia coli* (*E. coli*), as laid down in the European Bathing Water Directive (Directive 2006/7/EC). In the past few years, antibiotic-resistant bacteria such as extended spectrum beta-lactamase producing *E. coli* (ESBL-EC) and carbapenemase-producing *E. coli* (CP-EC) gained large interest. ESBL-EC are resistant toward third and fourth generation beta-lactam antibiotics but not against carbapenem antibiotics. They have been frequently found in effluents of municipal wastewater treatment plants (WWTPs),^10,11^ manure,^12,13^ surrounding areas of livestock buildings,^14,15^ surface waters,^16^ and sediments.^17^ CP-EC are of particular concern, as they are also resistant toward carbapenems, i.e., last resort antibiotics.^18^ In a nationwide study in The Netherlands, CP Enterobacterales (CPE) have been detected in 89 of 100 monitored WWTPs.^19^

Considerable efforts have already been undertaken to monitor *E. coli* in surface water and wastewater (e.g., refs (16), (19)). While these studies focus mainly on specific river sites downstream of known emission sources, some more comprehensive catchment-wide monitoring campaigns have been performed to quantify the impact of different emission sources, get insight in their environmental fate, and assess the status of fecal contamination (e.g., refs (20−22)). Catchment-wide studies on antibiotic-resistant bacteria (ARB), however, have rarely ever been carried out. Serwecińska et al. were the first to provide an overview of the occurrence of carbapenem-resistant *Acinetobacter* spp. on a catchment scale.^23^ For ESBL-EC and CP-EC, such comprehensive studies on the level of entire catchments do not exist.

Reaching conclusions beyond those from monitoring, fate modeling of bacteria in whole catchments can help with understanding underlying processes, comparing the importance of different emission sources, and identifying contamination hotspots. Especially when concentrations are close to or below the detection limit, e.g., with CP-EC given their rather low concentrations in WWTP effluent,^19^ predictive models can be useful tools to complement monitoring. The GREAT-ER (*Geography-Referenced Regional Exposure Assessment Tool for European Rivers*) model is well established for simulating chemical exposure in whole river catchments. It has been successfully applied to predict environmental concentrations of different chemicals like detergents,^24^ pharmaceuticals,^25−27^ and even dissolved zinc^28^ in various catchments. Recently, it has been applied for risk assessment of selected pharmaceuticals in the Dutch–German transboundary catchment of the Vecht River.^29^ The model covers processes such as emissions from WWTPs as point sources, in-stream transport, sedimentation, and degradation, in a steady state approach. While temperature and precipitation resolved monitoring is needed to identify risks during particular conditions (e.g., during overflows after heavy rains which can increase concentrations greatly), steady state models can be used to map gradients in average risks. In turn, this helps identify locations of lower and higher concern, which could be studied in more detail if needed.

The current study has two objectives. The first aim is to get insight into the spatiotemporal distribution and dynamics of (AR) *E. coli* in surface waters and wastewaters by combining a comprehensive one-year monitoring campaign in the catchment of the Dutch–German cross-border Vecht River with catchment modeling. We analyzed *E. coli*, ESBL-EC, and CP-EC in surface waters with and without WWTP influence as well as in WWTP influents and effluents. Then, the GREAT-ER model was extended to include a simulation routine for simulating (AR) *E. coli*. Predicted concentrations are evaluated against measured data. The second aim is to perform an exposure assessment during average weather conditions on the catchment scale based on predicted microbial concentrations.

## 2 Materials and Methods

### 2.1 Study Area

**Figure 1:**
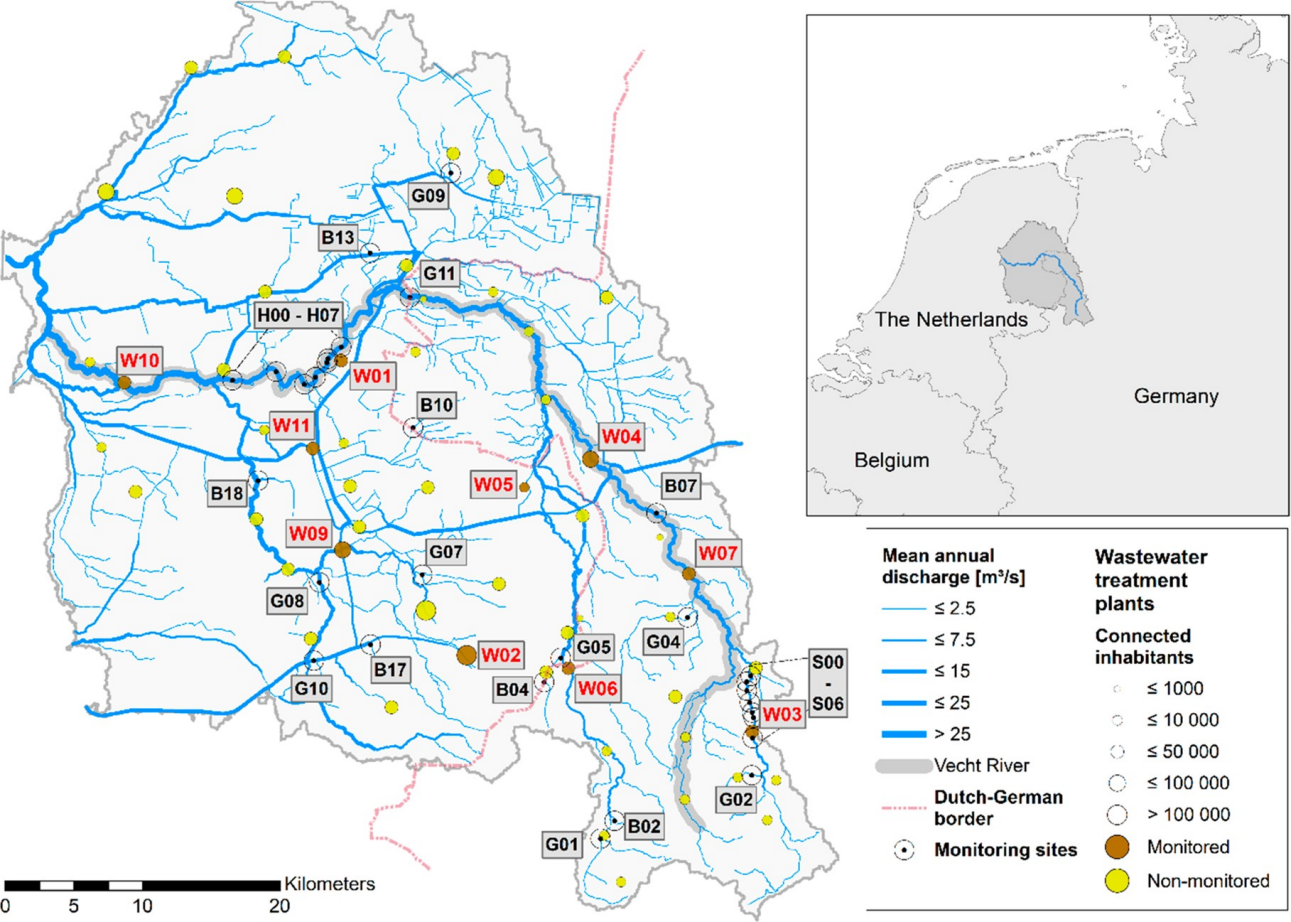
Overview map of the Vecht catchment area, monitoring sites, and monitored wastewater treatment plants (WWTPs). Letters in monitoring site IDs indicate different sampling site types: W, WWTP samples; B, background samples in areas without WWTP influence; S and H, longitudinal concentration profiles at WWTPs W03 and W01, respectively; G, general catchment samples.

The catchment area of the Dutch–German transboundary Vecht River, a tributary of the Dutch IJssel River, extends over an area of about 6100 km^2^ (Figure 1). Emissions from approximately 1.5 million inhabitants connected to 57 WWTPs enter the Vecht River or its tributaries. In addition, the wastewater of 13 hospitals is treated by these WWTPs. The catchment is characterized by high proportions of agricultural land use (Table S1). Further details are described in Duarte et al.^29^ and Wöhler et al.^30^

### 2.2 Monitoring Campaign

The monitoring campaign was run from July 2018 to August 2019 and included samples from 41 different locations (10 WWTPs and 31 surface water sites) spread over the entire Vecht catchment (Figure 1). Each location was sampled monthly with a maximum of 10 sampling moments. Exact coordinates of all sampling locations and sampling site IDs can be found in Table S2. All samples were analyzed for *E. coli* and ESBL-EC. CP-EC were only cultured from influent and effluent samples as in-stream concentrations were expected to be below the limit of quantification (LOQ). The sampling procedure and quantification methods can be found in the Supporting Information (Text 1, Sampling Procedure; Text 2, Quantification Methods). In short, concentrations of *E. coli*, ESBL-EC, and CP-EC were determined by enumeration based on selective agar plates. TBX agar was used for isolation of *E. coli*, Chromagar ESBL for ESBL-EC, and ChromID CARBA for non-OXA-48 CPE.^19^ Membrane filtration of a range of water volumes was used to generate bacterial concentrations.^31^ Five to 10 isolates from each sample were species confirmed by indole testing, and for a selection of isolates, phenotypic ESBL resistance and species identity were confirmed by VITEK-MS (BioMérieux, Amersfoort, The Netherlands).

Ten WWTPs were selected for monitoring on the basis of the plant location (Germany, 4; Netherlands, 6), scale (9000–180 000 inhabitants), and whether the plant treated hospital wastewater (5) or not (see Table S3). WWTP influent and effluent samples were collected at the same time. All WWTPs use conventional conventional activated sludge (CAS) treatment; two WWTPs have a hybrid treatment system combining CAS treatment with advanced treatment techniques.

Surface water sampling sites were selected to deliver data for three different situations, namely, longitudinal concentration profiles downstream of WWTPs W01 (NL) and W03 (GE), background locations without known WWTP effluent emissions (background samples B), and evaluation data from sites across the whole catchment with at least one WWTP emission upstream (general catchment samples G). Longitudinal profile samples included one reference site located upstream of the WWTPs W01 (H00) and W03 (S00) and several downstream sites over distances of 16.7 km (H01–H07) and 9.0 km (S01–S06), respectively (Table S2). No other point emissions are known to occur within these distances. The data were used to investigate (i) the local influence of WWTP emissions on *E. coli* concentrations and (ii) temporal variation along a longitudinal gradient. Background sites were selected as representative of diffuse bacterial sources with a focus on the effect of agricultural land use determined by the help of land use maps (cropland and pasture, Table S4). General catchment locations (G02–G11) are affected by at least one WWTP emission. Criteria for the selection of sampling sites were their proximity to gauging sites and their spatial distribution in the catchment to provide a useful basis for evaluation of the GREAT-ER model simulations.

In total, 196 WWTP influent and effluent samples, 70 background samples, 72 and 62 samples for the longitudinal profiles W01 and W03, respectively, and 90 general catchment samples were collected and analyzed (Tables S5, S6).

### 2.3 Evaluation of Wastewater Treatment Plant Samples

Assuming constant *E. coli* loads per person, measured influent and effluent concentrations are transformed into per capita loads (pcL_B,**m**_ [CFU cap^–1^ d^–1^]), similarly to Pallares-Vega et al.^32^ Data on the number of inhabitants connected to the WWTPs as well as discharges on the sampling days were provided by the respective authorities and water boards (Table S7).
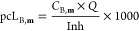
1*C*_B,**m**_ [CFU L^–1^] is the concentration of bacteria B (*E. coli*, ESBL-EC, or CP-EC) in sampling matrix **m** (influent or effluent). *Q* [m^3^ d^–1^] is the WWTP discharge of the respective day. Inh [cap] is the number of connected inhabitants, and 1000 is the unit conversion factor from cubic meters to liters. It was assumed that WWTP discharge was the same for the influent and effluent at a given date.

WWTP treatment efficiency (bacterial reduction logRed_B_) was calculated from the logarithms of influent (*C*_B,in_ [CFU L^–1^]) and effluent concentrations (*C*_B,eff_ [CFU L^–1^]) of the respective bacteria B:^33^
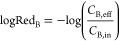
2

Since detection frequencies of CP-EC in effluents were too low, reduction was calculated for *E. coli* and ESBL-EC only.

Relative abundances of AR *E. coli* (*r*_ARB,**m**_) in the influent and effluent (matrices **m**) are calculated by normalizing their concentrations with total *E. coli* concentrations, following published approaches.^34,35^
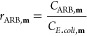
3We evaluated the contribution of different factors on *E. coli* influent loads, log reduction, and relative abundance of AR *E. coli* (ESBL-EC and CP-EC) to total *E. coli* using linear mixed models, with WWTP as a random factor to correct for clustering of observations within WWTPs. Response variables and explanatory variables are displayed in Table S8. The analysis was performed in R (version 3.6.3) using packages lme4 and lmerTest.^36,37^ The final models were inspected for heteroscedasticity and homogeneity of variances.

### 2.4 Modeling Microbial Water Quality in the Vecht Catchment

#### 2.4.1 The GREAT-ER Model

The GREAT-ER model is a spatially resolved river catchment model following a mass balance approach and assuming steady state conditions. The model was originally developed to predict aquatic exposure concentrations of down-the-drain chemicals in entire catchments.^38,39^ Contaminants are traversed as loads through the river network with a spatial resolution of 2 km flow length. Final concentrations are derived by dividing simulated loads with the flow rate of the respective river segment. A conceptual representation of the model is provided in the Supporting Information (Figure S1). The GREAT-ER software is implemented as an add-in for the geographic information system ESRI ArcGIS, versions 10.0 and higher. Technical details are provided in Kehrein et al.^26^ and Lämmchen et al.^38^

#### 2.4.2 Hydrological Representation of the Vecht Catchment

The Vecht catchment is characterized by strong anthropogenic influence on the hydrological conditions. Especially in The Netherlands, a network of canals has been installed to keep water levels in the Vecht River constant in summer for year-round navigability. Additionally, tributaries are prevented from falling dry by pumping IJssel water into the area. This makes the usual stochastic description of the natural flow rate variation virtually impossible. Instead, two different scenarios describing season-specific typical hydrologic conditions were created for the Vecht catchment. A detailed description of the setup and the hydrological representation is provided by Lämmchen et al.^25^ Its general applicability was recently confirmed by a case study with pharmaceuticals.^25,29^ The first scenario represents the situation of dry weather in summer (dry summer scenario), and the second one describes humid periods throughout the whole year (average flow scenario), where the flow rate is affected by interflow and surface runoff due to precipitation (see Table S9 for more details).

#### 2.4.3 Emission Estimation

Average daily excretion of *E. coli* per person is assumed constant. *E. coli* emissions from WWTPs are thus modeled analogous to pharmaceuticals assuming a constant per capita emission rate pcL_in_ [CFU cap^–1^ d^–1^] coupled with the logarithmic reduction efficiency of bacterial loads by wastewater treatment (logRed [-]):
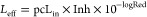
4*L*_eff_ [CFU d^–1^] is the daily effluent load, and Inh [cap] is the number of inhabitants connected to the WWTP.

The contribution of different processes to *E. coli* concentrations in rural areas was investigated earlier, e.g., by applying the SWAT model.^6,40,41^ However, due to a lack of quantitative information about relevant parameters (e.g., sediment concentrations, wildlife coverage, groundwater exchange, and runoff data across the whole catchment), diffuse emissions of *E. coli* are modeled by a simple empirical approach summarizing all contributions in one parameter, namely a constant concentration *C*_B_ [CFU L^–1^] in background inflow. Local emission loads (*I*_B_ [CFU d^–1^]) for each river section are generated by multiplying this concentration with the flow increment between two adjacent river segments Δ*Q* [m^3^ s^–1^]:
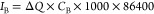
51000 and 86 400 are conversion factors from cubic meters to liters and from days to seconds.

Parameterization of WWTP and diffuse emissions was based on measured microbial concentrations from WWTP and background monitoring sites (see section 2.4.5).

#### 2.4.4 Fate of *E. coli* in Surface Water

Due to the small size, the settling velocity of free *E. coli* bacteria in rivers is very slow. Efficient settling only occurs when the bacteria are attached to suspended material.^42^ Sedimentation of *E. coli* thus depends on the fraction of bacteria attached to suspended particles (*f*_s_) and the settling velocity (*v*_set_ [m h^–1^]) of these particles. The settling velocity of 0.1 m h^–1^ is adopted from the *E. coli* study in the Scheldt catchment.^43^ Sedimentation is then parametrized as a first order process with settling rate *k*_set_ [h^–1^] and water depth *d* [m]:^44^
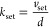
6

Specific information about the attachment behavior of *E. coli* in the Vecht catchment was not available. Therefore, we aggregated data on *E. coli* attachment to suspended particles observed in other catchments resulting in a median fraction of 36.5% (Table S10). The same parameters were also used to simulate ESBL-EC and CP-EC concentrations.

Survival of *E. coli* in surface waters depends on several environmental factors, including water temperature, solar radiation, pH, salinity, nutrient availability, and predation.^45,46^ Among them, water temperature is commonly regarded as the major factor.^47^ Temperature-dependent inactivation (die-off) of free floating *E. coli* can be described by a first order rate constant *k*(ϑ) [h^–1^],^47,48^ which is temperature corrected according to Ouattara et al.:^43^
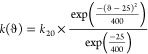
7ϑ [°C] is the temperature, and *k*_20_ [h^–1^] is the inactivation rate at 20 °C. The temperature correction has been successfully applied to explain the dynamics of fecal coliforms in the catchment of the Scheldt river for *k*_20_ set to 4.5 × 10^–2^ h^–1^.^43^ Temperatures for the average flow scenario (11.9 °C) and the dry summer scenario (18.2 °C) were extracted from daily measurements in the catchment in the years 2016–2019. Inactivation rates of ESBL-EC and CP-EC were assumed the same as for *E. coli*. Inactivation rates of bacteria associated with suspended particles are set to 50% of the rate for free floating bacteria.^49^

#### 2.4.5 Model Training and Performance Evaluation

Data from WWTP samples and background samples were used for the parametrization of emissions. WWTP samples were evaluated with respect to seasonal and national differences to estimate average values for pcL_in_ and logRed in the two scenarios (for more details, see Supporting Information, Text S3 with Tables S11–S13). Average *E. coli* background concentrations (*C*_B_) were estimated by fitting observed concentrations at background sites to the model (for more details, see Supporting Information, Text S4 with Table S14). For both WWTP and diffuse emissions, AR bacteria emissions were estimated on the basis of observed relative abundances. Sampling site G10 is used for model parametrization of loads entering the Vecht catchment from the IJssel via the Twente Canal.

Model performance was evaluated by comparing predicted with measured surface water concentrations from general catchment samples and longitudinal profile samples. For the latter, only the upstream sampling locations (S00 and H00) and the sampling locations farthest downstream (S06 and H07) were included to avoid bias from correlated data within longitudinal profiles. Summer measurements (i.e., June 21 to September 22) were allocated to the dry summer scenario and the remaining samples to the average flow scenario. Since measured bacterial concentrations range over several orders of magnitude, the evaluation was performed on log-transformed data. GREAT-ER simulations reflect steady state without temporal resolution and variation caused by differences in, e.g., flow and temperature. Thus, monitoring data at individual sites were aggregated into median values for comparison, whereby analysis data below the LOQ were processed as the respective concentration.

The coefficient of determination (*R*^2^) and the percentage bias (PBIAS) were used as model performance metrics for *E. coli* and ESBL-EC separately for each of the two scenarios. This was not possible for CP-EC because it was not analyzed in surface water samples (see section 2.2). *R*^2^ describes the proportion of the variance in the measured data which can be explained by the model and is widely used for the evaluation of water quality models.^50^ Statistical significance (*p* value) of *R*^2^ was examined by calculating the F statistic. PBIAS indicates if predicted concentrations are rather overestimate (PBIAS > 0) or underestimate (PBIAS < 0) observed concentrations.^50^

### 2.5 Human Exposure Assessment

Surface waters are being used for recreational purposes, not only at designated bathing sites that are regularly monitored.^24,57^ The Vecht catchment in particular is frequently used for recreational purposes including swimming.^58^ The *E. coli* threshold value for good bathing water quality in terms of fecal contamination is defined in the Bathing Water Directive as 10 000 CFU L^–1^. For the exposure assessment, we assume waterbodies with an average depth of at least 0.5 m to be potential swimming sites and evaluate predicted *E. coli* concentrations against the threshold at these sites for both scenarios. Since no such threshold exists for AR *E. coli*, we estimate the amount taken up during a single swimming event based on a swallowed water volume of 18 and 27 mL for women and men, respectively.^59^

## 3 Results and Discussion

### 3.1 Monitoring Overview

Log-transformed *E. coli* concentrations in WWTP influents were 7.92 ± 0.35 log CFU L^–1^ and 5.21 ± 0.74 log CFU L^–1^ in effluents. ESBL-EC loads in influent and effluent wastewater were approximately 2–3 log units below respective *E. coli* concentrations, namely, 5.97 ± 0.47 and 3.25 ± 0.71 log CFU L^–1^, respectively (Figure S2). All values are in the range of previously reported data, e.g., refs (11, 19, and 51). *E. coli* concentrations in background surface water samples were about two orders of magnitude lower than effluent concentrations (3.10 ± 0.85 log CFU L^–1^), and general catchment samples were slightly higher contaminated (3.46 ± 0.81 log CFU L^–1^). The detection frequency of ESBL-EC in surface water was 67% and 39% for general catchment and background sites, respectively. CP-EC was only detected in 43% of influent and 16% of effluent samples, and concentrations were lower than for ESBL*-*EC. Summary statistics are provided in the Supporting Information (Tables S15–S19).

### 3.2 Analysis of Wastewater Samples with Linear Mixed Models

*E. coli* per capita influent loads amounted to pcL_*E.coli*,influent_ = (2.2 ± 1.8) × 10^10^ CFU cap^–1^ d^–1^. According to linear mixed models on data from all WWTPs, the *E. coli* influent load was independent of WWTP discharge and country but showed significantly higher values in summer (*p* < 0.001, Tables S11 and S12).

Log *E. coli* reduction in the investigated WWTPs (2.68 ± 0.9) was in the upper region of reduction values reported for conventional wastewater treatment (between 1 and 3 log units).^10,11,32,51−53^ No difference between *E. coli* and ESBL-EC removal was observed (p ≫ 0.1). Effluent samples showed larger variation in per capita loads, both for *E. coli* and ESBL-EC, but no apparent seasonal trend of effluent concentrations was recognizable (Figure S3). Linear mixed modeling showed that log reduction was inversely correlated with normalized WWTP discharge as a proxy for rainfall (*p* < 0.001), probably due to lower residence time in the WWTP at a higher discharge.^32^

Relative abundance of ESBL-EC (ESBL-EC to *E. coli* ratio) was significantly higher in the German WWTPs (factor 2 on average) compared to those in The Netherlands (*p* < 0.001). To the best of our knowledge, this is the first study showing national differences in relative ESBL-EC abundance in wastewater, albeit on a regional scale. Higher ESBL-EC abundances may be attributed to differences in prevalence between countries. Approximately 5% of the Dutch population carries ESBL-EC.^19^ In Germany, a prevalence of 6.8% was reported in 2015^54^ and 2.3% in 2015–2017,^55^ albeit both were determined with a slightly less sensitive method.^54,55^ Comparing the Dutch–German border region, no difference between the countries had been found earlier for the ESBL prevalence at hospital admittance.^56^ Our findings suggest a difference in community prevalence in the cross-border region of the Vecht catchment, which might have been left unnoticed in the population studies due to differences in methodology, or due to the focus on specific populations such as hospital patients.

Relative abundance of ESBL-EC was 0.14 log units (40%) higher in summer than in the remaining year (*p* < 0.001). Seasonal effects on ESBL-EC carriage have been found in population studies of ESBL population prevalence, possibly related to travel to non-European countries or environmental exposure (e.g., due to recreational activities) as risk factors for increased ESBL carriage in summer.^54,57^

Concentrations of CP-EC relative to *E. coli* in the influent of positive samples were on the same order of magnitude (10^–5^) as reported by Blaak et al.^19^ For relative CP-EC abundance, neither country nor season showed a significant effect.

### 3.3 Temporal Variation in Longitudinal Concentration Profiles

**Figure 2:**
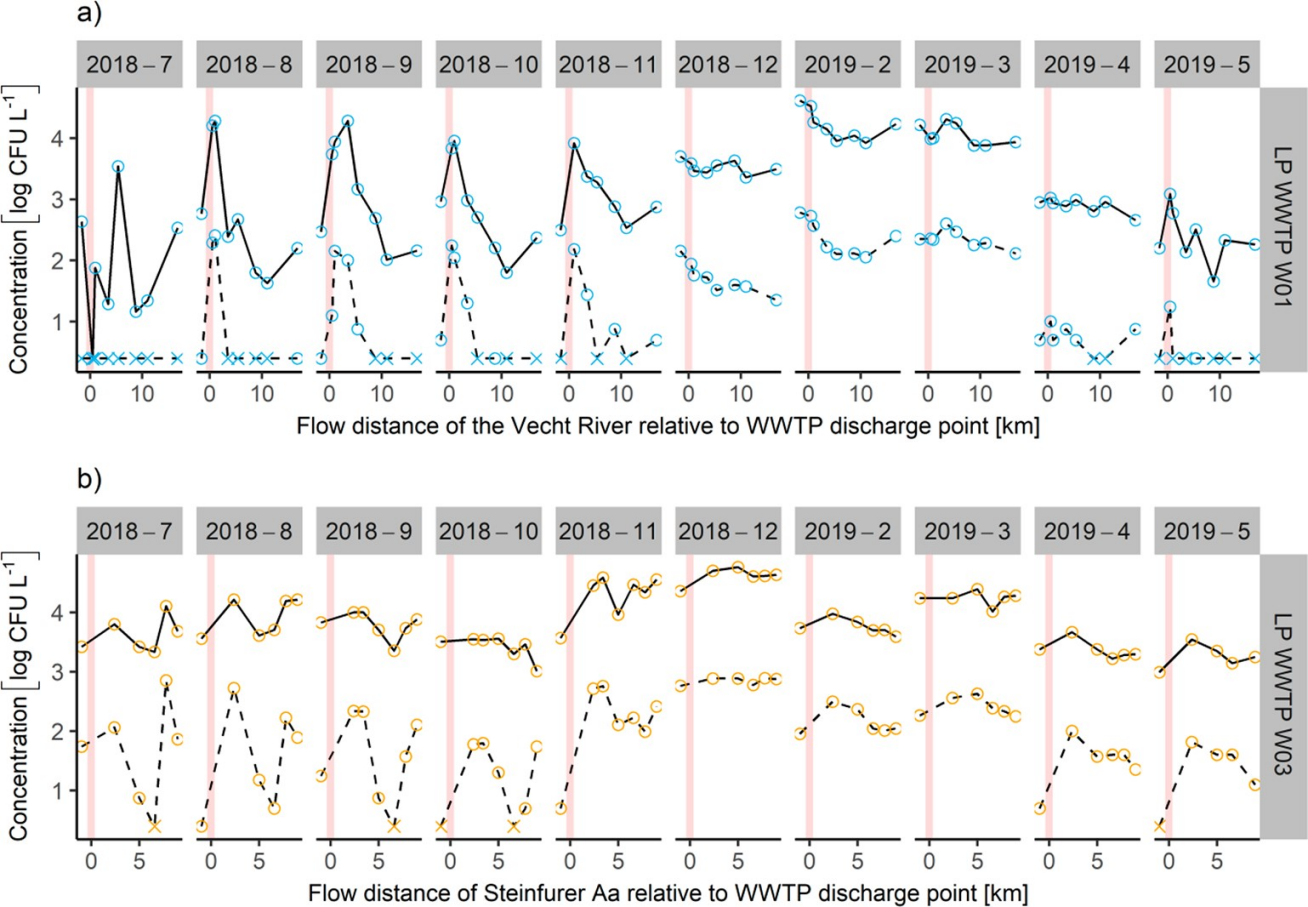
Measured concentrations of *E. coli* (solid lines) and ESBL *E. coli* (dashed lines) at longitudinal profiles (LPs) relative to discharge points of WWTPs W01 (a) and W03 (b). Crosses indicate concentrations below LOQ displayed as LOQ. Red lines indicate WWTP discharge points, i.e., relative flow distance of 0 km.

The local impact of WWTP emissions on downstream *E. coli* concentrations in receiving rivers has been documented in several studies, e.g., refs (51) and (58). Our data corroborate the local impact of WWTP effluent on downstream *E. coli* concentrations (see Figure 2), but the effect does not consistently occur at all sites and time points. The W01 profiles (Figure 2a) show no measurable effect of WWTP emission on *E. coli* and ESBL-EC concentrations at three of the 10 time points (December 2018 and February–March 2019). In February and March, the Vecht River at W01 exhibited high flow rates (three times the long-term annual average) together with high upstream concentrations. The latter might have been caused by stormwater runoff or combined sewage overflows (CSO), where *E. coli* concentrations have been reported to be up to 2 orders of magnitude higher than in the WWTP effluent in this study.^59,60^ Contributions from diffuse runoff can also significantly increase in-stream concentrations of *E. coli* after rain events.^20,61^ One process behind this can be remobilization of bacteria from the sediments by turbulent mixing at high flow rates.^20^ In July, monitoring data showed inexplicable profile dynamics at the longitudinal profile W01.

*E. coli* concentrations upstream of WWTP W03 (Figure 2b) are generally higher (on average by factor 3.6) than upstream of WWTP W01. Downstream of the WWTP, a concentration increase of 0.36 ± 0.28 log units is observed (Figure 2b). This effect is even more pronounced for ESBL-EC with an increase of 1.08 ± 0.75 log units, most likely because the relative abundance of AR bacteria in the diffuse background is lower compared to WWTP effluents. This effect is particularly large in months July–November 2018 and April–May 2019. At the sampling point 6.6 km downstream of the WWTP, concentrations almost consistently increase again, which hints at an unknown point source. For the sampling months December 2018 and February and March 2019, the WWTP emission effect is less clear.

### 3.4 Overall Model Evaluation

**Figure 3:**
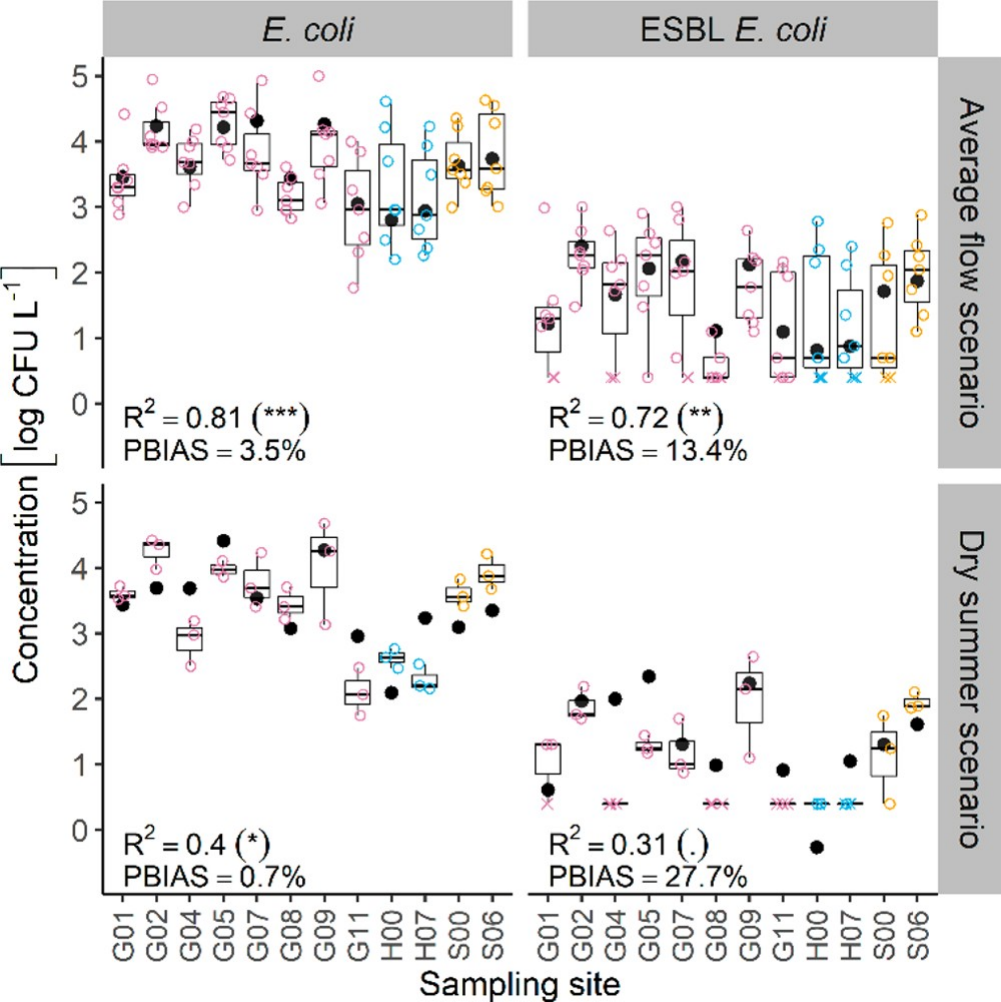
Measured and predicted concentrations of *E. coli* and ESBL *E. coli* bacteria in surface water at general catchment sites and longitudinal profiles (upstream and most distant point of longitudinal profile). Measured concentrations are displayed as box-whisker plots and predicted concentrations at steady state as solid black dots. Crosses indicate outliers. Symbols in brackets indicate significance levels of *R*^2^: ***p < 0.001, **p < 0.01, *p < 0.05, *. p* ≥ 0.05.

Simulated concentrations well explained the observed spatial variability of measured concentrations (represented by the spatial median) in the average flow scenario for *E. coli* (*p* < 0.001) and ESBL-EC (*p* < 0.01; Figure 3). In the dry summer scenario, prediction accuracy was weaker (less significant R^2^) for both *E. coli* and ESBL-EC. Interestingly, simulations applying individual on-site monitoring data for WWTP emissions did not increase the prediction accuracy compared to the assumption of average per-capita loads.

However, simulations showed a tendency toward overestimation (PBIAS > 0); e.g., in the longitudinal profile of WWTP W01, concentrations were overestimated. Overestimations were higher for ESBL-EC. ESBL-EC emissions are based on observed ESBL-EC abundances in WWTPs and at background sites relative to *E. coli* (Supporting Information, Text S3 and S4). ESBL-EC abundances below LOQ were not considered for parametrization of background sites, which may have introduced a bias toward higher input assumptions. Different in-stream inactivation and sedimentation rates for *E. coli* and ESBL-EC are unlikely, since ESBL-gene carriage has not been found to influence the fitness of *E. coli*.^62,63^

We conclude that application of generalized parameters to predict *E. coli* and ESBL-EC concentrations is feasible for aquatic exposure assessment in the Vecht catchment. Especially in the average flow scenario, the model correctly depicts spatial variations. Under dry weather conditions, better understanding of the contribution of diffuse emission processes and respective model refinement are necessary to increase the accuracy of the predictions.

### 3.5 Human Exposure Assessment in the Vecht Catchment

Human exposure assessment in the Vecht catchment is conducted only for potential swimming sites (average depth ≥ 0.5 m). This includes 44% of cumulated flow length (sum of the length of all simulated river segments in the Vecht catchment) in the average flow scenario. In the dry summer scenario, river depth is generally lower, and only waterbodies downstream of WWTPs are deep enough for bathing, making up 26% of cumulated flow length. Result maps for both scenarios are presented in Figures S4–S6. At the potential swimming sites, predicted *E. coli* concentrations range from less than 1 up to 113 000 CFU L^–1^ for both scenarios. Swimming in fecally contaminated water poses a risk of becoming infected by ingesting fecal pathogens such as the human norovirus.^64^ High *E. coli* concentrations indicate an increased risk of such an infection. The threshold value for a good bathing water quality (10 000 CFU L^–1^) laid down in the EU Bathing Water Directive is exceeded by predicted concentrations in only 6% and 7% of potential swimming waters for the average flow scenario and the dry summer scenario, respectively. Exceedance occurs mainly in rivers, where microbial loads emitted by WWTPs are not sufficiently diluted by the receiving waters. The overall contribution of *E. coli* emissions from WWTP effluents to total *E. coli* emissions in the Vecht catchment was 76% and 71% in the average flow scenario and the dry summer scenario, respectively. Due to the emission of *E. coli* by consecutive WWTPs within the river network predicted concentrations can exceed the threshold over up to 20 km flow length. This underlines the importance of WWTPs as a source of fecal contamination not only locally but also on the catchment scale. However, diffuse sources can be locally important as the impact of WWTP emissions decreases with increasing distance to the discharge point (Supporting Information, Text 5: Impact of Modeled Processes on *E. coli* Concentrations).

Predicted ESBL-EC concentrations in the Vecht catchment were approximately 2 to 3 orders of magnitude lower than *E. coli* concentrations in both scenarios, which is a direct consequence of assuming constant ESBL-EC/*E. coli* ratios for diffuse as well as for WWTP emissions (Supporting Information, Text S3 and S4). The WWTP effluent contribution to ESBL-EC emissions in the Vecht catchment was 96% in both scenarios. From the highest predicted ESBL-EC concentrations at potential swimming sites (1071 CFU L^–1^) in the average flow scenario, a theoretical uptake of 29 and 19 CFU per swimming event was derived for men and women, respectively. In the dry summer scenario, predicted concentrations reach up to 2272 CFU L^–1^, which translates into a higher potential uptake of 61 CFU and 41 CFU per swimming event, respectively.

CP-EC are assumed to be present exclusively downstream of WWTPs. This covers 37% of cumulated flow length in the Vecht catchment in the average flow scenario including the Vecht River and its main tributaries Steinfurter Aa, Dinkel, and Regge. In summer, water is pumped into smaller tributaries to avoid them falling dry (section 2.4.2). As a result, a larger fraction of cumulated flow length (53%) is affected by WWTP emissions in the dry summer scenario. CP-EC concentrations are difficult to quantify in surface waters due to their low concentrations. Modeling enables estimation of human exposure toward CP-EC during recreational activities such as swimming: Exposure to the highest predicted CP-EC concentration of 1.2 CFU L^–1^ (calculated in the dry summer scenario) would amount to an uptake of less than one CFU of CP-EC for both men and women. The theoretical human ingestion values however cannot directly be translated to a public health risk as dose–response relationships of ESBL-EC and CP-EC are lacking.^65^

### 3.6 Model Limitations and Recommendations for Future Investigations

Monitoring showed that diffuse input of *E. coli* and probably also of ESBL *E. coli* contribute to the overall contamination. Due to insufficient knowledge on the relative contributions of runoff, remobilization from the sediments and groundwater exchange to bacterial in-stream concentrations, these inputs were modeled with a simplified approach. Targeted investigations of *E. coli* and AR *E. coli* concentrations, especially in soil, sediment, and the different flow components, are required to get further insight for model refinement. Monitoring also revealed that WWTP per capita emission rates of (AR) *E. coli* vary by up to 1 order of magnitude between different WWTPs, which has also been found in other studies.^22,66^ The average WWTP emission rates applied in the model simulation proved to deliver a realistic overall picture but lead to over- or underestimation of local inputs. Investigation of the effect of different treatment steps and technologies on *E. coli* removal could help refine the model.

Sedimentation has a strong impact on predicted bacterial concentrations (Supporting Information, Text 5: Impact of Modeled Processes on *E. coli* Concentrations). The model assumes a constant fraction of *E. coli* attached to suspended matter. However, a range of 20–53.6% has been reported (Table S10). Partitioning of *E. coli* depends on the particle size^67^ and the clay content.^42^ The role of the suspended solid concentration has been used in many models to estimate the fraction of attached bacteria.^40,41,68,69^ However, these require detailed data about the composition of suspended solids in the Vecht catchment throughout the year. The sedimentation process itself depends on the settling velocity of the particles, where values between 5 × 10^–2^ and 1.05 m h^–1^ have been reported.^42^

The GREAT-ER model provides a realistic picture of the spatial concentration distribution across the catchment for standard flow scenarios but does not capture concentration variability visible from the monitoring data, since it assumes a temporal steady state. Thus, it also does not represent (i) the short-term effect of event-driven inputs such as surface runoff or CSO events and (ii) natural variability of model parameters. For example, *E. coli* inactivation rates derived from 95% of in-stream temperatures in the Vecht catchment (2.8–21.9 °C) lead to inactivation rates between 1.4 × 10^–2^ and 4.7 × 10^–2^ h^–1^. However, the model allows for identification of locations of higher-than-average risk. Natural variability and uncertainty of input parameters can be considered in GREAT-ER by applying the already implemented stochastic Monte Carlo simulation routine. An appropriate representation for Monte Carlo simulations is currently parametrized in a German subcatchment of the Vecht River. This allows the prediction of expected ranges of microbial concentrations over the course of a year and to systematically investigate the sensitivity of parameters on predicted concentrations.

## Supporting Information Available

Sections on sampling procedure, quantification methods, model parametrization and calibration, and impact of modeled processes on *E. coli* concentrations along with supporting tables and figures (PDF)

## Accession Codes

The concentrations of *E. coli*, ESBL-producing *E. coli*, and CP-*E. coli* measured in the Dutch–German Vecht catchment are available in the DANS repository, 10.17026/dans-xpy-h27s. A basic version of the GREAT-ER model is publicly available (https://www.usf.uni-osnabrueck.de/en/forschung/applied_systems_science/great_er_project). The full version of the model generated during the current study and the source code is available from the author Jörg Klasmeier on reasonable request and against registration.
