# Supplementary material for: (Antibiotic-Resistant) E. coli in the Dutch–German Vecht Catchment—Monitoring and Modeling
Source: Environ Sci Technol. 2022 Jun 3;56(21):15064–73. doi: 10.1021/acs.est.2c00218 (PMC9631988; doi:10.1021/acs.est.2c00218)
Supplement: Supplementary file 1 — es2c00218_si_001.pdf [file es2c00218_si_001.pdf]

Supplementary Information

(Antibiotic resistant) *E. coli* in the Dutch-German Vecht catchment – Monitoring and modeling

Authors: Eri van Heijnsbergen [a,1], Gunnar Niebaum [b,1], Volker Lämmchen [b], Alicia Borneman [a], Lucia Hernández Leal [a], Jörg Klasmeier [b,2], Heike Schmitt *[a,c,2]

[a] Wetsus, European Centre of Excellence for Sustainable Water Technology, Oostergoweg 9, 8911 MA Leeuwarden, the Netherlands,

[b] Institute of Environmental Systems Research, Osnabrück University, Barbarastraße 12, D-49076, Osnabrück, Germany, [c] Institute for Risk Assessment Sciences, Utrecht University, Yalelaan 2, 3584 CM Utrecht, the Netherlands

[1] These authors contributed equally as first authors to this work.

[2] These authors contributed equally as senior authors to this work.

* Corresponding author at: Wetsus, European Centre of Excellence for Sustainable Water Technology, Oostergoweg 9, 8911 MA Leeuwarden, the Netherlands. E-mail address: heike.schmitt@wetsus.nl

Pages: 33

Text 1: Sampling procedure

Text 2: Quantification methods

Text 3: WWTP model parametrization

Text 4: Background sites – monitoring and model parametrization

Text 5: Impact of modeled processes on *E. coli* concentrations

Contains: Supplementary Figures (S1 – S9) and Supplementary Tables (S1 – S19)

Table S1. Land use in the Vecht catchment. Data is summarized from CORINE Land Cover [1].

Land use	Share	Comment
Arable land	27.1%	Non-irrigated arable land
Forest	9.7%	
Grasland, shrubs, transitional land	1.5%	
Mixed land use	17.7%	Principally consists of cultivated areas and pastures with areas of natural vegetation and scattered houses or gardens
Pastures	33.2%	Pastures, meadows and other permanent grasslands under agricultural use
Surface waters	0.3%	
Urban	9.5%	Cities, roads, industry, roads, rail networks, airports, mineral extraction sites and dump sites
Wetland	1.0%	

Text 1: Sampling procedure

WWTP operators provided 24-h samples of WWTP influent and effluent. Some of the plants provided flow proportional samples. Surface water samples were taken according to NEN 6600-2 [2] using a sampling stick with 1 L beaker or a 10 L bucket dropped down from a bridge. Sampling time, weather, water temperature and circumstances were noted. Samples were cooled during transportation, stored at 4 °C and processed within 24 hours.

Text 2: Quantification methods

For isolation of *E. coli*, ESBL-producing E. coli (ESBL-EC) and carbapenemase-producing *E. coli* (CP-EC), water samples were filtered through a membrane filter with a pore size of 0.45 µm (Merck, Amsterdam, the Netherlands) according to ISO 8199:2018 [3]. Different dilutions and volumes (ranging from 10 mL to 300 mL) were used depending on the expected bacterial concentration of the different sample types.

After filtration, the filters were placed on selective agar plates and incubated for 4 hours at 37 °C and 18-24 hours at 44 °C. To quantify *E. coli*, Tryptone Bile X-glucuronide agar (TBX) (EWC Diagnostics, Steenwijk, The Netherlands; ref. T703.02) was used in accordance with ISO 16649–2 [4]. For quantification of resistant bacteria, the following agar plates were used: ChromID ESBL (BioMérieux, Amersfoort, the Netherlands; ref. 43481) to detect ESBL-EC and ChromID CARBA (BioMérieux; ref. 43861) to detect CP-EC (i.e. focus was laid on expected CP-EC genotypes other than OXA-48). All samples were analyzed for E. coli and ESBL-EC, while CP-EC were only cultured from influent and effluent samples. From the counts, concentrations in CFU L-1 were calculated according to ISO 8199 [3], except that counts with a total number of less than 10 colonies were also included for ESBL-EC and CP-EC. From every ESBL and CARBA plate, 5 – 10 colonies were confirmed with an indole test for species identity. A selection of the colonies was also subjected to species identification and confirmation of phenotypic ESBL resistance by VITEK (BioMérieux, Amersfoort, the Netherlands) and combination disk test (https://www.eucast.org/fileadmin/src/media/PDFs/EUCAST_files/Resistance_mechanisms/EUCAST_detection_of_resistance_mechanisms_170711.pdf). 92 out of 92 tested isolates (100%) from ChromID ESBL plates showed a phenotype indicative of extended spectrum beta lactamase (ESBL) production. A selection of CP-EC (192) was also subjected to WGS for confirmation of species and CP gene carriage.

Table S2. Sampling site coordinates of the monitoring campaign.

Sample type	Sampling site ID	Coordinates (Latitude, Longitude)	Relative to WWTP (only longitudinal profile)
WWTP	W01	N52°32'27.48", E6°36'23.41"	
WWTP	W02	N52°14'02.8", E6°50'36.8"	
WWTP	W03	N52° 9'46.95", E7°20'2.09"	
WWTP	W04	N52°26'43.89", E7° 2'43.88"	
WWTP	W05	N52°24'43.9", E6°55'46.7"	
WWTP	W06	N52°13'27.2", E7°00'49.9"	
WWTP	W07	N52°19'35.9", E7°13'13.0"	
WWTP	W09	N52°20'28.9", E6°37'31.6"	
WWTP	W10	N52°30'37.3", E6°14'14.5"	
WWTP	W11	N52°26'48.6", E6°33'56.0"	
Longitudinal profile	H00	N52°33'22.55", E6°36'33.17"	1.6 km upstream
Longitudinal profile	H01	N52°32'36.22", E6°35'13.93"	0.5 km downstream
Longitudinal profile	H02	N52°32'21.23", E6°35'8.39"	1.0 km downstream
Longitudinal profile	H03	N52°31'23.88", E6°34'4.31"	3.5 km downstream
Longitudinal profile	H04	N52°30'55.14", E6°32'51.51"	5.4 km downstream
Longitudinal profile	H05	52°30'45.61", E6°30'53.80"	8.8 km downstream
Longitudinal profile	H06	N52°31'40.48", E6°29'54.87"	11.0 km downstream
Longitudinal profile	H07	N52°31'1.43", E6°25'25.47"	16.8 km downstream
Longitudinal profile	S00	N52° 9'21.51", E7°20'10.01"	1.0 km upstream
Longitudinal profile	S01	N52°10'39.61", E7°20'13.40"	2.4 km downstream
Longitudinal profile	S02	N52°10'57.56", E7°20'0.50"	3.4 km downstream
Longitudinal profile	S03	N52°11'36.30", E7°19'46.01"	5.0 km downstream
Longitudinal profile	S04	N52°12'20.67", E7°19'26.93"	6.6 km downstream
Longitudinal profile	S05	N52°12'54.03", E7°19'27.01"	7.8 km downstream
Longitudinal profile	S06	N52°13'16.75", E7°19'49.16"	9.0 km downstream
Background site	B02	N52° 3'55.81", E7° 6'11.57"	
Background site	B04	N52°12'33.99", E6°58'35.98"	
Background site	B07	N52° 23'25.72", E7°9'40.89"	
Background site	B10	N52°28'24.25", E6°44'15.97"	
Background site	B13	N52°39'22.76", E6°39'16.59"	
Background site	B17	N52°14'35.84", E6°40'36.44"	
Background site	B18	N52°24'43.51", E6°28'26.56"	

Table S2 continued. Sampling site coordinates of the monitoring campaign.

Sample type	Sampling site ID	Coordinates (Latitude, Longitude)	Relative to WWTP (only longitudinal profile)
General catchment site	G01	N52° 2'46.76", E7° 4'49.56"	
General catchment site	G02	N52° 7'1.37", E7°20'9.30"	
General catchment site	G03	N52°13'16.75", E7°19'49.16"	
General catchment site	G04	N52°16'52.76", E7°13'10.75	
General catchment site	G05	N52°14'7.75", E7° 0'12.35"	
General catchment site	G07	N52°19'6.48", E6°45'42.91"	
General catchment site	G08	N52°18'25.62", E6°35'8.79"	
General catchment site	G09	N52°44'35.29", E6°47'22.29"	
General catchment site	G10	N52°13'27.40", E6°34'48.60"	
General catchment site	G11	N52°36'38.35", E6°43'31.05"	

Table S3. Characteristics of the ten monitored wastewater treatment plants (WWTPs) in the Vecht catchment

Sampling site ID	Country	Connected population	Receiving hospital wastewater	Advanced treatment techniques	Nr of sampling events
W01	NL	32 050	Yes		10
W02	NL	179 917	Yes		10
W03	GE	14 712			9
W04	GE	128 300	Yes		10
W05	NL	9 233		Hybrid MBR[b]	10
W06	GE	47 269	Yes		9
W07	GE	30 600			10
W09	NL	95 167	Yes		10
W10	NL	26 390			10
W11	NL	18 550		Hybrid Nereda[c]	10

[a] WWTPs that for downstream concentration profile measurement.
[b] Up to 50% of receiving wastewater is treated by a membrane bio-reactor.
[c] A parallel operating activated sludge system is fed with Nereda® waste sludge which settles more easily

Table S4. Land use upstream of background sites. Land use classes refer to Table S1.1.

Monitoring site	Arable land	Pastures	Mixed land use	Forest	Other land use classes
B02	85.3%	5.9%	0.0%	6.3%	2.5%
B04	33.4%	37.1%	3.5%	7.7%	18.3%
B07	77.8%	6.5%	0.0%	15.6%	0.1%
B10	58.2%	10.6%	0.0%	25.3%	5.9%
B13	68.5%	12.0%	5.4%	0.0%	14.1%
B17	7.2%	21.4%	50.9%	18.8%	1.7%
B18	5.4%	55.3%	25.2%	2.2%	11.9%

Table S5. Sampling dates of all WWTP samples. On every sampling date, both an influent and an effluent sample were taken. Eight WWTPs were sampled from July 2018 on and sampling of another two WWTPs started in December 2018. In January 2019, due to practical circumstances, no samples were taken except for the German WWTPs. In the months February to May 2019, some WWTPs were not able to provide samples for a variety of reasons. These WWTPs provided an extra sample in the month June.

Sampling date	W09	W10	W02	W06	W01	W04	W05	W07	W03	W11
2018-07-18	2	0	0	0	2	2	2	2	0	2
2018-07-23	0	2	0	0	0	0	0	0	0	0
2018-07-25	0	0	2	0	0	0	0	0	0	0
2018-08-07	0	2	2	0	0	0	2	0	0	0
2018-08-14	0	0	0	0	2	0	0	0	0	2
2018-08-22	0	0	0	0	0	2	0	2	0	0
2018-08-30	2	0	0	0	0	0	0	0	0	0
2018-09-10	0	0	0	0	0	0	0	2	0	0
2018-09-11	0	2	0	0	2	2	0	0	0	2
2018-09-18	2	0	2	0	0	0	2	0	0	0
2018-10-09	0	2	0	0	2	0	0	0	0	2
2018-10-17	0	0	0	0	0	2	0	2	0	0
2018-10-18	2	0	2	0	0	0	2	0	0	0
2018-11-06	2	0	2	0	0	0	2	0	0	0
2018-11-13	0	2	0	0	2	0	0	0	0	2
2018-11-14	0	0	0	0	0	2	0	2	0	0
2018-12-04	0	2	0	0	2	0	0	0	0	2
2018-12-05	0	0	0	2	0	2	0	2	0	0
2018-12-10	0	0	0	0	0	0	0	0	2	0
2018-12-13	2	0	2	0	0	0	2	0	0	0
2019-01-09	0	0	0	2	0	2	0	2	2	0
2019-02-07	2	0	2	0	0	0	2	0	0	0
2019-02-13	0	2	0	0	2	0	0	0	0	0
2019-02-18	0	0	0	0	0	0	0	0	2	0
2019-02-20	0	0	0	2	0	2	0	2	0	0
2019-03-05	0	2	0	0	2	0	0	0	0	2
2019-03-06	0	0	0	0	0	0	0	0	2	0
2019-03-11	0	0	0	2	0	2	0	2	0	0
2019-03-14	2	0	0	0	0	0	2	0	0	0
2019-04-04	0	2	0	0	2	0	0	0	0	2
2019-04-09	0	0	2	0	0	0	2	0	0	0
2019-04-10	0	0	0	0	0	0	0	0	2	0
2019-04-15	0	0	0	2	0	2	0	2	0	0
2019-05-06	0	0	0	0	0	0	0	0	2	0
2019-05-14	2	0	2	0	0	0	2	0	0	0
2019-05-15	0	0	0	2	0	0	0	0	0	0
2019-05-21	0	0	0	0	2	0	0	0	0	2
2019-06-25	2	2	2	2	0	0	0	0	2	2
2019-07-22	0	0	0	2	0	0	0	0	2	0
2019-08-19	0	0	0	2	0	0	0	0	2	0

Table S6. Sampling dates of surface water samples (background, general catchment samples and longitudinal profiles).

Sampling date	B02	B04	B07	B10	B13	B17	B18	G01	G02	G04	G05	G07	G08	G09	G10	G11	W01 Profile	W03 Profile
2018-07-16	0	1	1	0	0	0	0	0	0	0	1	0	0	1	0	0	0	0
2018-07-18	0	0	0	1	0	1	1	0	0	0	0	1	1	0	1	0	0	0
2018-07-23	0	0	0	0	1	0	0	0	0	0	0	0	0	0	0	1	8	0
2018-07-25	1	0	0	0	0	0	0	1	1	1	0	0	0	0	0	0	0	6
2018-08-06	0	1	1	0	0	0	0	0	0	0	1	0	0	1	0	0	0	0
2018-08-13	0	0	0	1	0	1	1	0	0	0	0	1	1	0	1	0	0	0
2018-08-15	0	0	0	0	1	0	0	0	0	0	0	0	0	0	0	1	8	0
2018-08-22	1	0	0	0	0	0	0	1	1	1	0	0	0	0	0	0	0	6
2018-09-10	0	1	1	0	0	0	0	0	0	0	1	0	0	1	0	0	0	0
2018-09-12	0	0	0	1	0	1	1	0	0	0	0	1	1	0	1	0	0	0
2018-09-19	1	0	0	0	0	0	0	1	1	1	0	0	0	0	0	0	0	7
2018-09-26	0	0	0	0	1	0	0	0	0	0	0	0	0	0	0	1	8	0
2018-10-08	0	0	0	0	1	0	0	0	0	0	0	0	0	0	0	1	8	0
2018-10-10	0	0	0	1	0	1	1	0	0	0	0	1	1	0	1	0	0	0
2018-10-15	1	0	0	0	0	0	0	1	1	1	0	0	0	0	0	0	0	7
2018-10-22	0	1	1	0	0	0	0	0	0	0	1	0	0	1	0	0	0	0
2018-11-05	0	0	0	1	0	1	1	0	0	0	0	1	1	0	1	0	0	0
2018-11-07	0	0	0	0	1	0	0	0	0	0	0	0	0	0	0	1	7	0
2018-11-12	1	0	0	0	0	0	0	1	1	1	0	0	0	0	0	0	0	7
2018-11-14	0	1	1	0	0	0	0	0	0	0	1	0	0	1	0	0	0	0
2018-12-03	0	0	0	1	0	1	1	0	0	0	0	1	1	0	1	0	0	0
2018-12-05	0	1	1	0	0	0	0	0	0	0	1	0	0	1	0	0	0	0
2018-12-10	1	0	0	0	0	0	0	1	1	1	0	0	0	0	0	0	0	6
2018-12-12	0	0	0	0	1	0	0	0	0	0	0	0	0	0	0	1	8	0
2019-02-11	0	0	0	0	1	0	0	0	0	0	0	0	0	0	0	1	8	0
2019-02-13	0	0	0	1	0	1	1	0	0	0	0	1	1	0	1	0	0	0
2019-02-18	1	0	0	0	0	0	0	1	1	1	0	0	0	0	0	0	0	6
2019-02-20	0	1	1	0	0	0	0	0	0	0	1	0	0	1	0	0	0	0
2019-03-06	0	0	0	0	0	0	0	1	1	1	0	0	0	0	0	0	0	6
2019-03-11	0	1	1	0	0	0	0	0	0	0	1	0	0	1	0	0	0	0
2019-03-13	1	0	0	1	0	1	1	0	0	0	0	1	1	0	1	0	0	0
2019-03-18	0	0	0	0	1	0	0	0	0	0	0	0	0	0	0	1	8	0
2019-04-01	0	0	0	0	1	0	0	0	0	0	0	0	0	0	0	1	8	0
2019-04-03	0	0	0	1	0	1	1	0	0	0	0	1	1	0	1	0	0	0
2019-04-10	1	0	0	0	0	0	0	1	1	1	0	0	0	0	0	0	0	6
2019-04-15	0	1	1	0	0	0	0	0	0	0	1	0	0	1	0	0	0	0
2019-05-06	1	0	0	0	0	0	0	1	1	1	0	0	0	0	0	0	0	5

Table S6 continued. Sampling dates of surface water samples (background, general catchment samples and longitudinal profiles).

Sampling date	B02	B04	B07	B10	B13	B17	B18	G01	G02	G04	G05	G07	G08	G09	G10	G11	W01 Profile	W03 Profile
2019-05-08	0	0	0	0	1	0	0	0	0	0	0	0	0	0	0	1	8	0
2019-05-13	0	0	0	1	0	1	1	0	0	0	0	1	1	0	1	0	0	0
2019-05-15	0	1	1	0	0	0	0	0	0	0	1	0	0	1	0	0	0	0

Table S7. Wastewater treatment plant (WWTP) data providers.

WWTP	Data providers
W01	Waterschap Vechtstromen
W02	Waterschap Vechtstromen
W03	LANUV [a], Kreisstadt Steinfurt
W04	NLWKN [b], Kommunale Betriebe Nordhorn
W05	Waterschap Vechtstromen
W06	LANUV [a], Stadtwerke Gronau
W07	NLWKN [b], Kommunale Betriebe Nordhorn
W09	Waterschap Vechtstromen
W10	Waterschap Drents Overijsselse Delta
W11	Waterschap Vechtstromen

[a] Lower Saxony Water Management, Coastal Defence and Nature Conservation Agency

[b] State Agency for Nature, Environment and Consumer Protection

Table S8. Response variables and explanatory variables used in the linear mixed models.

Response variables [a]	Explanatory variables						
	Country (Netherlands, Germany)	Seasons (summer, remaining year) [b]	Normalized WWTP discharge [c]	Bacteria (*E. coli*, ESBL-EC) [d]	Hospital wastewater (yes, no)	Matrix (influent, effluent)	WWTP (random factor)
$pcL_{E.\ coli,\ In}$	X	X	X				X
logRed	X	X	X	X			X
$r_{ESBL\text{-}EC}$	X	X			X	X	X
$r_{CP\text{-}EC}$	X	X			[e]	[d]	X

[a] Response variables: $pcL_{E.\ coli,\ In}$: per capita influent load of *E. coli*, *logRed*: reduction of bacteria during wastewater treatment, $r_{EBSL\text{-}EC}$: relative abundance of ESBL *E. coli* in wastewater, $r_{CP\text{-}EC}$: relative abundance of *CP E. coli* in wastewater.

[b] Summer in the Northern Hemisphere: June 21 – September 22.

[c] Normalized by dry weather flow

[d] Detection frequency of CP-EC in WWTP effluents was too low to calculate CP-EC reduction or to account for possible matrix effects.

[e] Detection frequency of CP-EC was too low in WWTPs not treating hospital effluents.

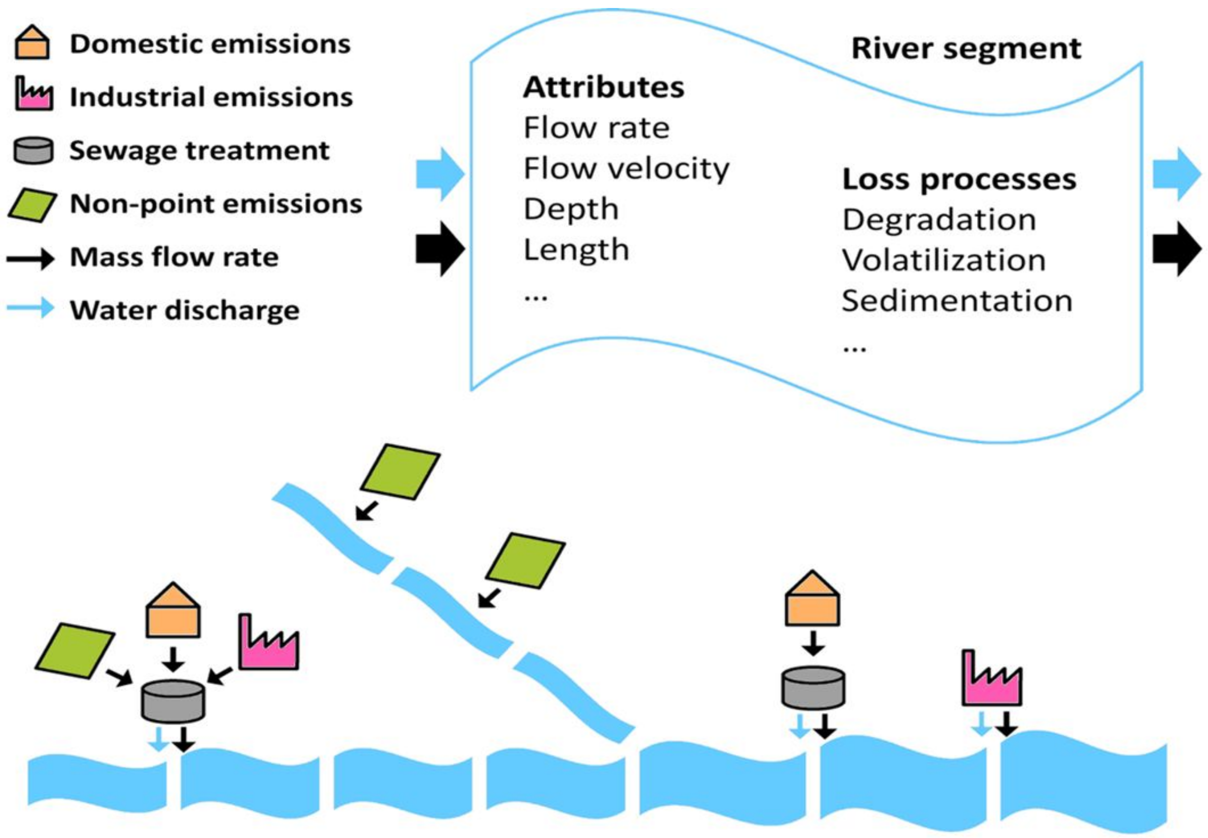

Figure S1. Conceptual representation of the GREAT-ER model. Concentrations are calculated by dividing mass flow rates by the water discharge of a river segment.

Table S9. Characteristics of the average flow scenario and the dry summer scenario (adopted from Duarte et al. (2020)[5])

	Average flow scenario	Dry summer scenario
Applicability	Humid periods throughout the whole year	Dry periods without rainfall between June and September
Flow rate at the border [$m^3 s^{-1}$]	18.5	2.8
Flow rate at the Zwarte Water [$m^3 s^{-1}$]	63.5	11.3
Pumping activity	No	Yes
Average water temperature [°C]	11.9	18.2

Table S10. Fractions of *E. coli* attached to suspended materials in natural waterbodies. Median attached fraction: 36.5%.

Attached fraction [%]	Source	Matrix
34.0	[6]	Suspended sediment
20.0	[6]	Suspended sediment
44.0	[6]	Suspended sediment
27.0	[6]	Suspended sediment
30.0	[7]	Suspended matter
36.5	[8]	Suspended solids
37.5	[8]	Suspended solids
53.6	[8]	Suspended solids
38.0	[9]	Suspended particles

Text 3: WWTP model parametrization

Different parameter sets were used for the average flow scenario and the dry summer scenario depending on boundary conditions, e.g. season or country. Parameters were derived from statistical analysis with linear mixed models (Section 2.3). Final reduced models are presented in Table S11 and parametrization for the reduced models in Tabel S12. Resulting input parameters for the GREAT-ER model are presented in Table S13. For the model evaluation (Section 3.4), all WWTPs are parametrized by the same per capita influent load and WWTP reduction; i.e. monitored WWTPs are parametrized in the same way as non-monitored WWTPs to evaluate the applicability of a generalized model. For the exposure assessment (Section 3.5) however, monitored WWTPs are parametrized based on on-site measurement data to include best available information.

To illustrate the WWTP model parametrization an example is provided: Calculation of *E. coli* loads in WWTP effluents in WWTP W01 (32 050 inhabitants) for the dry summer scenario. Daily effluent loads (L_{eff} [CFU d^{-1}]) are calculated as

$$L_{eff} = pcL_{in} \cdot Inh \cdot 10^{-logRed}$$

Where pcL_{in} is the per capita influent load [CFP cap^{-1} d^{-1}] of *E. coli, Inh* [cap] is the number of inhabitants connected to the WWTP and *logRed* is the logarithmic reduction of bacterial loads by wastewater treatment. pcL_{in} of *E. coli* is calculated with model 2 (Table S11):

$$\log_{10} pcL_{in} = 10.206 + 0.352 \cdot \mathrm{X}_1$$

where X_1 is 1 for summer and 0 for the remaining year. This results in $\log_{10} pcL_{in}$ = 10.558 and leads to pcL_{in} = 3.61 x 10^{10} CFU cap^{-1} d^{-1}. *logRed* is calculated with model 4 (Table S11):

$$logRed = 3.142 + 0.305 \cdot X_1 - 0.399 \cdot X_2$$

where X_1 is 1 for summer and 0 for the remaining year and X_2 is the normalized WWTP discharge, i.e. discharge normalized by dry weather flow (DWF). This indicates that treatment efficiency is highest,

when WWTP discharge is low and when it is summer. For the dry summer scenario we assume that the discharge is equal to the DWF for all WWTPs in summer. This leads to

$$logRed = 3.142 + 0.305 \cdot 1 - 0.399 \cdot 1 = 3.048$$

The fully parametrized WWTP emission model for *E. coli* in the dry summer scenario for WWTP W01 is then:

$$L_{eff} = 3.61 \cdot 10^{10} \cdot 32\ 050 \cdot 10^{-3.048} = 1.04 \cdot 10^{12}$$

In the dry summer scenario, WWTP W01 is predicted to release 1.04 x 10^{12} CFU of *E. coli* per day into the receiving river.

Table S11. Linear mixed models. Full and reduced models. Reduced models only consist of variables that were found to be significant in the full models. Acronyms and abbreviations: log.load: *E. coli* influent load per inhabitant. Season: divided into summer and remaining year. Country: Germany and the Netherlands. WWTP: Monitored wastewater treatment plant. log.red: log_{10} reduction of bacteria in WWTP. Bacteria: *E. coli* and ESBL *E. coli* (ESBL-EC); detection frequency of carbapenemase producing *E. coli* (CP-EC) was too low in effluent to calculate removal efficiencies. log.ratio.esbl: log_{10} of ESBL-EC to *E. coli* ratio. log.ratio.cpec: log_{10} of CP-EC to *E. coli* ratio. Hospital: WWTP treats hospital wastewater. Matrix: Influent and effluent.

Variable	Model number	Model type	Model formula
E. coli influent	1	Full	log.load ~ Season + Country + Q.norm + (1|WWTP)
	2	Reduced	log.load ~ Season + (1|WWTP)
Removal	3	Full	log.red ~ Season + Country + Q.norm + Bacteria + (1|WWTP)
	4	Reduced	log.red ~ Season + Q.norm + (1|WWTP)
ARB ratio	5	Full	log.ratio.esbl ~ Season + Country + Hospital + Matrix + (1|WWTP)
	6	Reduced	log.ratio.esbl ~ Season + Country + (1|WWTP)
	7	Full	Log.ratio.cpec ~ Season + Country + (1|WWTP)
	8	Reduced	Log.ratio.cpec ~ (1|WWTP)

Table S12. Parametrization of reduced linear mixed models. Acronyms and abbreviations: log.load: log_{10} of *E. coli* per capita load in WWTP influent. Season: divided into summer and remaining year. Country: Germany (GE) and the Netherlands (NL). WWTP: Monitored wastewater treatment plant. log.red: log_{10} reduction of bacteria in WWTP. log.ratio.esbl: log_{10} of ESBL-EC to *E. coli* ratio. log.ratio.cpec: log_{10} of CP-EC to *E. coli* ratio.

Model number	Model formula	Factor	Intercept/Beta	SE	p	CI lower	CI upper
2	log.load ~ Season + (1|WWTP)	Intercept	10.206	0.036	< 0.001	10.129	10.283
		Season summer	0.352	0.048	< 0.001	0.258	0.446
4	log.red ~ Season + Q.norm + (1|WWTP)	Intercept	3.142	0.188	<0.001	2.745	3.541
		Season summer	0.305	0.097	< 0.01	0.115	0.495
		Q.norm	-0.399	0.047	< 0.001	-0.491	-0.307
6	log.ratio.esbl ~ Season + Country + (1|WWTP)	Intercept	-1.796	0.055	< 0.001	-1.914	-1.678
		Season summer	0.139	0.042	< 0.001	0.057	0.221
		Country NL	-0.324	0.069	< 0.001	-0.475	-0.174
8	Log.ratio.cpec ~ (1|WWTP)	Intercept	-5.291	0.190	<0.001	-5.813	-4.905

Table S13. GREAT-ER model input parameters. pcL_{in}: Per capita influent load, logRed: log_{10} reduction of bacteria in WWTP. GE: Germany, NL: Netherlands.

Parameter	Bacteria	Scenario	Country	Value	Comment
pcL_{in}	*E. coli*	Average flow scnenario	GE, NL	1.61×10^{10} [CFU cap^{-1} d^{-1}]	Calculated with model 2
pcL_{in}	ESBL *E. coli*	Average flow scnenario	GE	2.57×10^{8} [CFU cap^{-1} d^{-1}]	ESBL *E. coli* to *E. coli* ratio calculated with model 6 is applied to per capita load of *E. coli* calculated with model 2
pcL_{in}	ESBL *E. coli*	Average flow scnenario	NL	1.22×10^{8} [CFU cap^{-1} d^{-1}]	ESBL *E. coli* to *E. coli* ratio calculated with model 6 is applied to per capita load of *E. coli* calculated with model 2
pcL_{in}	CP *E. coli*	Average flow scnenario	GE, NL	8.22×10^{4} [CFU cap^{-1} d^{-1}]	CP *E. coli* to *E. coli* ratio calculated with model 8 is applied to per capita load of *E. coli* calculated with model 2
pcL_{in}	*E. coli*	Dry summer scenario	GE, NL	3.61×10^{10} [CFU cap^{-1} d^{-1}]	Calculated with model 2
pcL_{in}	ESBL *E. coli*	Dry summer scenario	GE	7.96×10^{8} [CFU cap^{-1} d^{-1}]	ESBL *E. coli* to *E. coli* ratio calculated with model 6 is applied to per capita load of *E. coli* calculated with model 2
pcL_{in}	ESBL *E. coli*	Dry summer scenario	NL	3.78×10^{8} [CFU cap^{-1} d^{-1}]	ESBL *E. coli* to *E. coli* ratio calculated with model 6 is applied to per capita load of *E. coli* calculated with model 2
pcL_{in}	CP *E. coli*	Dry summer scenario	GE, NL	1.85×10^{5} [CFU cap^{-1} d^{-1}]	CP *E. coli* to *E. coli* ratio calculated with model 8 is applied to per capita load of *E. coli* calculated with model 2
logRed	*E. coli*, ESBL *E. coli*, CP *E. coli*	Average flow scnenario	GE, NL	Each WWTP individually, depending on Q.norm: logRed = 3.142 – 0.399 x Q.norm [-]	Calculated with model 4; Q.norm is calculated as the ratio of average daily discharge to dry weather flow of the respective WWTP
logRed	*E. coli*, ESBL *E. coli*, CP *E. coli*	Dry summer scenario	GE, NL	3.048 [-]	Calculated with model 4; Q.norm is equal to 1

Text 4: Background sites – monitoring and model parametrization

E. coli concentrations in background samples ranged over almost four orders of magnitude (0.81 - 4.61 log CFU L^{-1} (median 3.17 log CFU L^{-1}). Due to lower concentrations – often close to or below the detection limit, the range of ESBL concentrations was smaller (< LOQ - 2.18 log CFU L^{-1}). The median concentration of positive samples was 0.70 log CFU L^{-1}. Relative abundance of ESBL-EC was lower in background sites (0.14%) as compared to WWTP effluents by approximately one order of magnitude. Three background locations (i.e., B02, B04, B07) have a relatively high *E. coli* concentration and detection rate of ESBL-EC compared to the other background sites, for which we could find no obvious reasons. [10] measured median *E. coli* and ESBL-EC concentrations of 1.5 x 10^3 and 5.7 CFU L^{-1}, respectively, with 32% of ESBL-EC above the LOQ during a 9-month sampling campaign at an agricultural monitoring site without WWTP influence.

In the model, bacterial concentrations C [CFU L^{-1}] in river flow increments ΔQ are defined to estimate the respective diffuse emission loads (Section 2.4.3). In a calibration step, these concentrations were adjusted so that measured concentrations at background sampling sites best agreed with the simulation results. This results in increment concentrations of 4.5 x 10^3 and 3.1 x 10^4 CFU L^{-1} in the average flow scenario and the dry summer scenario, respectively (Table S14).

Due to the large number of non-detects (see Figure S2), parametrization of the ESBL-EC increment concentrations was based on *E. coli* using relative abundance of ESBL-EC. We assume that ESBL-EC to *E. coli* ratios are always the same in all river flow increments. From measured data at the background monitoring sites a median value of 0.14% was derived for this ratio. For CP-EC no such data was available. Therefore, diffuse emissions of CP-EC were not considered.

For this study, diffuse emissions of bacteria are thought to encompass (i) passive transport by the flow components runoff, interflow, baseflow and (ii) remobilization of bacteria from the sediments. These processes are thought to contribute differently to diffuse emissions and background concentrations in the

two modeled scenarios. The exact quantification of the contribution of individual processes however, cannot be provided here due to insufficient data and process understanding.

In the dry summer scenario, where mainly groundwater exchange is responsible for river flow, diffuse emissions of bacteria are thought to mainly account for remobilization of bacteria from the sediments. Sediments are a reservoir for *E. coli* bacteria [11]. The work by Kim et al. (2010) indicates that sediment concentrations of *E. coli* bacteria are 2 – 3 orders of magnitude higher in summer and autumn compared to the remaining year [12]. The bacteria in the sediment reservoir can be mobilized by groundwater flowing into the river [13] but also by active mobilization [14]. Pachepsky et al. (2017) even observed an increase in *E. coli* concentrations under base flow conditions [13].

In the average flow scenario, the remobilization of bacteria from the sediments is thought to additionally appear due to bed shear stress due to high flows [6]. Considering the work of Kim et al. (2010) [12], (2 – 3 orders of magnitude higher *E. coli* concentrations in the sediments in summer and autumn), the total load entering the water column by remobilization is thought to be lower compared to the dry summer scenario. In contrast to the dry summer scenario, the flow in the average flow scenario also consists of interflow and runoff. Especially the latter contributes to diffuse emissions of *E. coli* in the Vecht catchment, which is characterized by agricultural activities. Due to the manure application on arable land and grassland as well as livestock on pasture land, fecal bacteria, i.e. *E. coli*, are introduced to agricultural areas. Here, they can survive several months before they are transported by surface flow or washed off into adjacent rivers [15].

Table S14. Parametrization of bacterial concentrations [CFU L^{-1}] in river flow increments *ΔQ*.

Scenario	*E. coli*	ESBL *E. coli*	CP *E. coli*
Dry summer	3.1×10^4	43.4	n.a.[a]
Average flow	4.5×10^3	6.3	n.a.[a]

[a] Not applied: Diffuse emissions are neglected for CP *E. coli* due to insufficient data.

Table S15. Descriptive statistics for *E. coli* concentrations in WWTP influents and effluents. DF: Detection frequency. n = 10 for all WWTPs, except for W03 and W06 (n = 9).

	Influent (log CFU L^{-1})					Effluent (log CFU L^{-1})				
Sampling site ID	Mean	Median	Min	Max	DF	Mean	Median	Min	Max	DF
W01	8.03	7.84	7.31	8.38	100%	6.15	6.12	5.64	6.52	100%
W02	8.14	8.09	7.55	8.48	100%	4.84	4.86	4.13	5.15	100%
W03	7.73	7.61	6.96	8.19	100%	5.25	4.85	4.06	5.86	100%
W04	8.15	8.03	7.55	8.48	100%	5.09	4.60	4.20	5.68	100%
W05	7.97	7.99	7.38	8.32	100%	5.58	4.52	3.83	6.36	100%
W06	7.90	7.76	7.26	8.26	100%	6.01	5.83	3.66	6.37	100%
W07	8.17	8.14	7.70	8.47	100%	4.82	4.69	3.96	5.38	100%
W09	8.02	8.03	6.69	8.30	100%	5.93	5.23	4.50	6.83	100%
W10	7.96	7.80	7.55	8.24	100%	5.45	5.21	3.66	6.00	100%
W11	8.06	8.00	7.61	8.34	100%	6.15	5.81	5.30	6.77	100%

Table S16. Descriptive statistics for ESBL *E. coli* concentrations in WWTP influents and effluents. DF: Detection frequency. n = 10 for all WWTPs, except for W03 and W06 (n = 9).

	Influent (log CFU L^{-1})					Effluent (log CFU L^{-1})				
Sampling site ID	Mean	Median	Min	Max	DF	Mean	Median	Min	Max	DF
W01	6.01	5.66	4.89	6.40	100%	3.95	3.84	3.20	4.36	100%
W02	6.36	6.30	5.54	6.79	100%	2.97	2.87	2.56	3.30	100%
W03	5.89	5.84	5.29	6.14	100%	3.49	3.09	2.40	4.05	100%
W04	6.47	6.37	5.78	6.86	100%	3.07	2.95	2.26	3.49	100%
W05	5.95	5.79	4.54	6.48	100%	3.24	2.42	1.36	3.99	100%
W06	6.05	5.86	5.47	6.46	100%	4.30	4.24	3.36	4.70	100%
W07	6.44	6.45	5.79	6.78	100%	3.05	2.97	2.53	3.40	100%
W09	6.17	6.17	4.80	6.47	100%	4.17	3.28	2.13	5.11	100%
W10	5.90	5.64	5.17	6.39	100%	3.34	3.09	1.66	3.99	90%
W11	6.07	5.75	5.44	6.55	100%	4.03	3.87	3.13	4.58	100%

Table S17. Descriptive statistics for CP *E. coli* concentrations in WWTP influents and effluents. DF: Detection frequency. n = 10 for all WWTPs, except for W03 and W06 (n = 9).

	Influent (log CFU L^{-1})					Effluent (log CFU L^{-1})				
Sampling site ID	Mean	Median	Min	Max	DF	Mean	Median	Min	Max	DF
W01	2.98	2.98	1.62	3.27	20%	1.10	1.10	1.10	1.10	10%
W02	3.54	2.76	1.62	4.21	90%	< LOQ	< LOQ	< LOQ	< LOQ	0%
W03	2.62	2.62	2.62	2.62	11%	0.70	0.70	0.70	0.70	11%
W04	3.26	2.70	1.62	3.81	90%	0.94	0.94	0.40	1.18	20%
W05	< LOQ	< LOQ	< LOQ	< LOQ	0%	< LOQ	< LOQ	< LOQ	< LOQ	0%
W06	3.28	3.22	1.92	3.68	89%	1.73	1.35	1.23	2.25	56%
W07	1.51	1.51	1.36	1.62	20%	< LOQ	< LOQ	< LOQ	< LOQ	0%
W09	3.40	3.00	2.10	3.94	90%	2.14	1.18	0.40	2.81	50%
W10	2.46	2.46	2.46	2.46	10%	1.00	1.00	1.00	1.00	10%
W11	1.62	1.62	1.62	1.62	10%	0.40	0.40	0.40	0.40	10%

Table S18. Descriptive statistics for bacterial concentrations at background sites. DF: Detection frequency. n = 10 for all sampling sites.

	E. coli (log CFU L^{-1})					ESBL *E. coli* (log CFU L^{-1})				
Sampling site ID	Mean	Median	Min	Max	DF	Mean	Median	Min	Max	DF
A02	3.91	3.89	3.27	4.28	100%	1.33	0.40	0.39	1.98	60%
A04	4.03	3.77	2.82	4.61	100%	1.43	1.05	0.39	2.18	80%
A07	4.04	3.74	3.02	4.53	100%	1.04	0.57	0.40	1.52	60%
A10	3.22	2.29	0.81	4.11	100%	1.10	1.18	0.40	1.30	30%
A13	2.77	2.65	2.23	3.26	100%	0.40	0.40	0.40	0.40	10%
A17	3.38	3.05	2.17	3.84	100%	1.49	1.49	0.40	1.78	20%
A18	2.46	2.28	1.37	2.95	100%	0.88	0.88	0.88	0.88	10%

Table S19. Descriptive statistics for bacterial concentrations at general catchment sites. DF: Detection frequency. n = 10 for all sampling sites.

	E. coli (log CFU L^{-1})					ESBL *E. coli* (log CFU L^{-1})				
Sampling site ID	Mean	Median	Min	Max	DF	Mean	Median	Min	Max	DF
G01	3.70	3.47	2.89	4.41	100%	2.20	1.30	1.18	2.99	70%
G02	4.35	4.03	3.91	4.95	100%	2.18	2.11	1.48	2.63	90%
G04	3.69	3.53	2.50	4.19	100%	2.22	2.09	1.72	2.64	50%
G05	4.32	4.06	3.72	4.68	100%	2.26	1.66	0.48	2.90	100%
G07	4.19	3.68	2.95	4.93	100%	2.12	1.87	0.70	2.81	80%
G08	3.34	3.26	2.82	3.71	100%	0.78	0.70	0.48	1.11	50%
G09	4.12	4.11	3.06	4.68	90%	2.17	2.00	1.11	2.64	100%
G10	3.05	2.09	1.36	3.75	100%	1.68	1.68	1.54	1.78	20%
G11	3.32	2.51	1.75	4.00	100%	1.78	1.66	0.48	2.16	60%

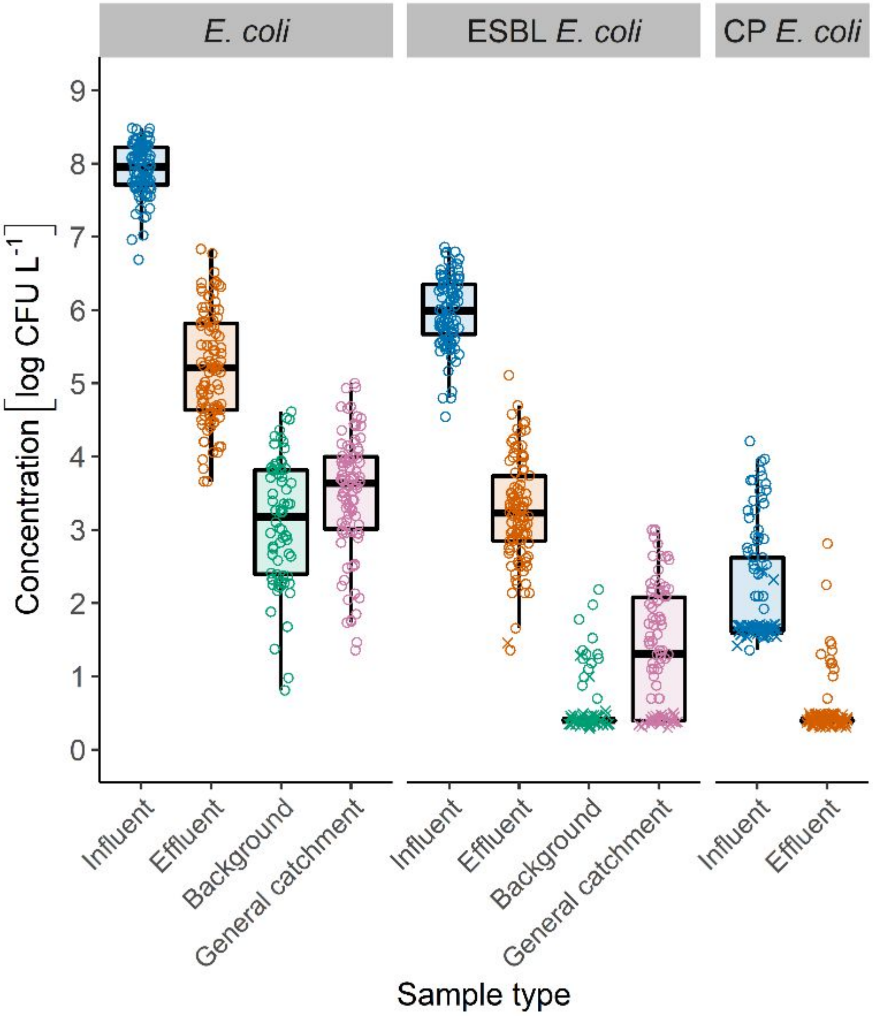

Figure S2. Measured concentrations of *E. coli*, ESBL *E. coli* and CP *E. coli* bacteria in wastewater (influent and effluent samples) and surface water (background samples, general catchment samples). Crosses indicate concentrations below LOQ displayed as LOQ.

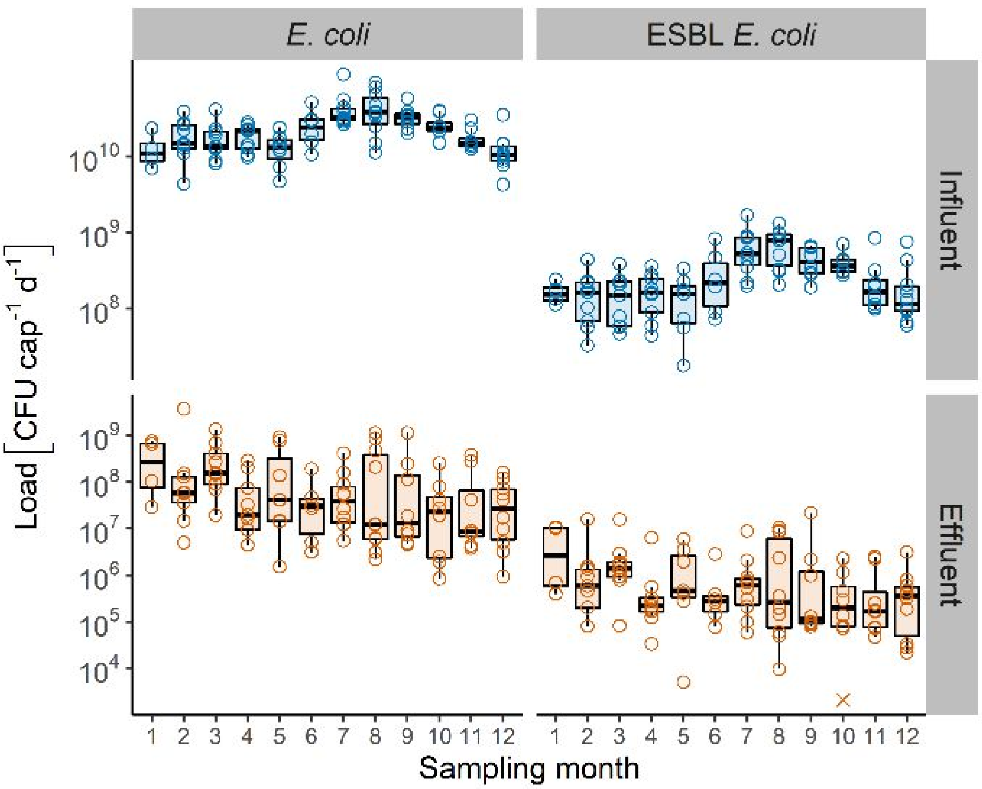

Figure S3. Measured loads of *E. coli* and ESBL *E. coli* bacteria in wastewater (influent and effluent samples) over time. Crosses indicate concentrations below LOQ displayed as LOQ. January – June: 2019. July – December: 2018. July and August include two samples each from 2019. Influent loads exhibit a temporal trend with higher values in the period between June and October – i.e. mainly in summer - for *E. coli* as well as for ESBL-EC.

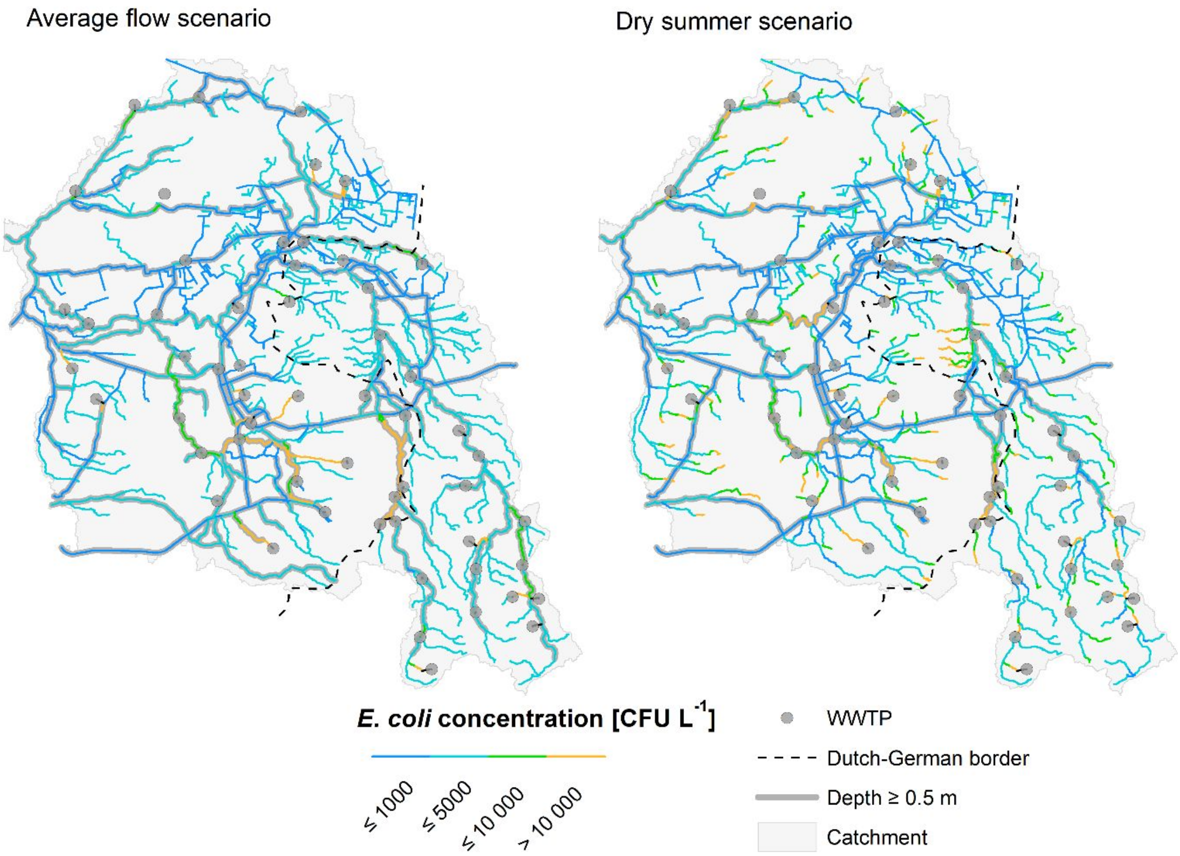

Figure S4. Predicted concentration of *E. coli* in the average flow and the dry summer scenario. WWTP = wastewater treatment plant. A depth ≥ indicates potential swimming sites.

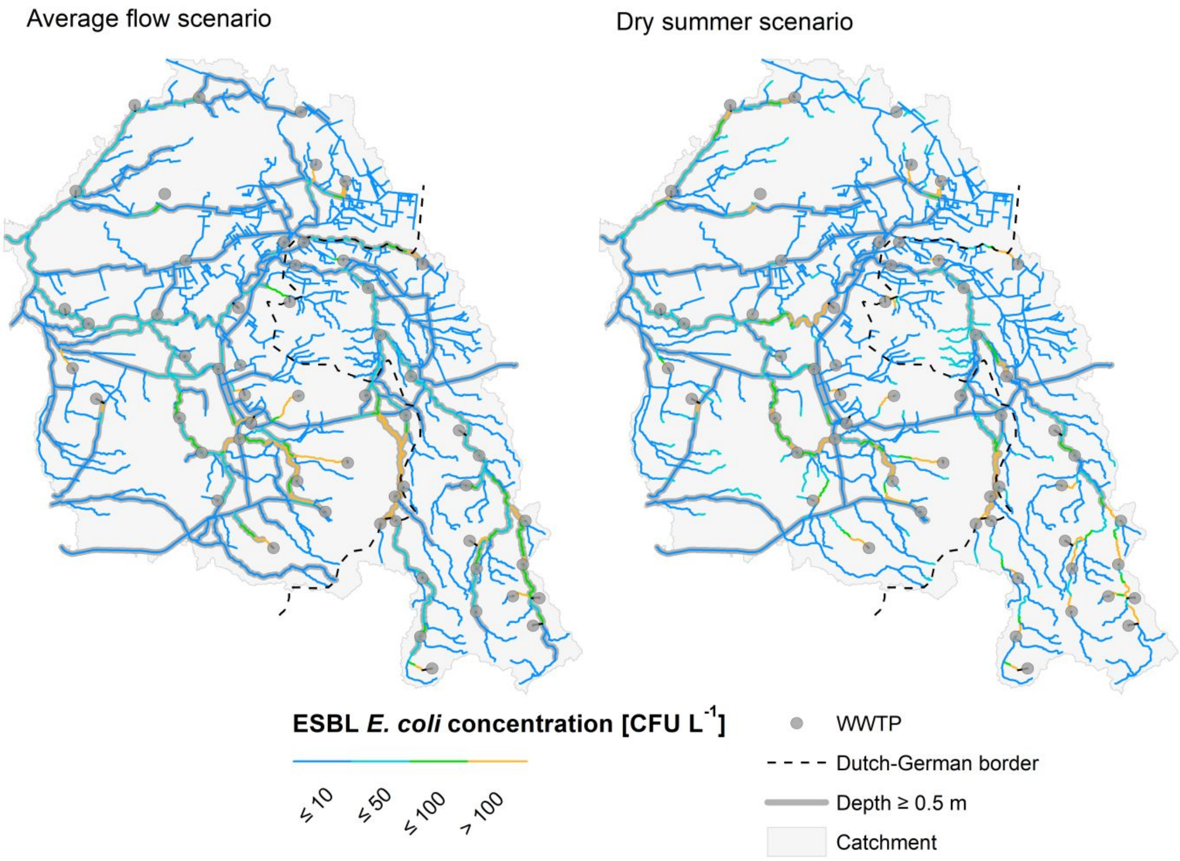

Figure S5. Predicted concentration of ESBL *E. coli* in the average flow and the dry summer scenario. WWTP = wastewater treatment plant. A depth ≥ indicates potential swimming sites.

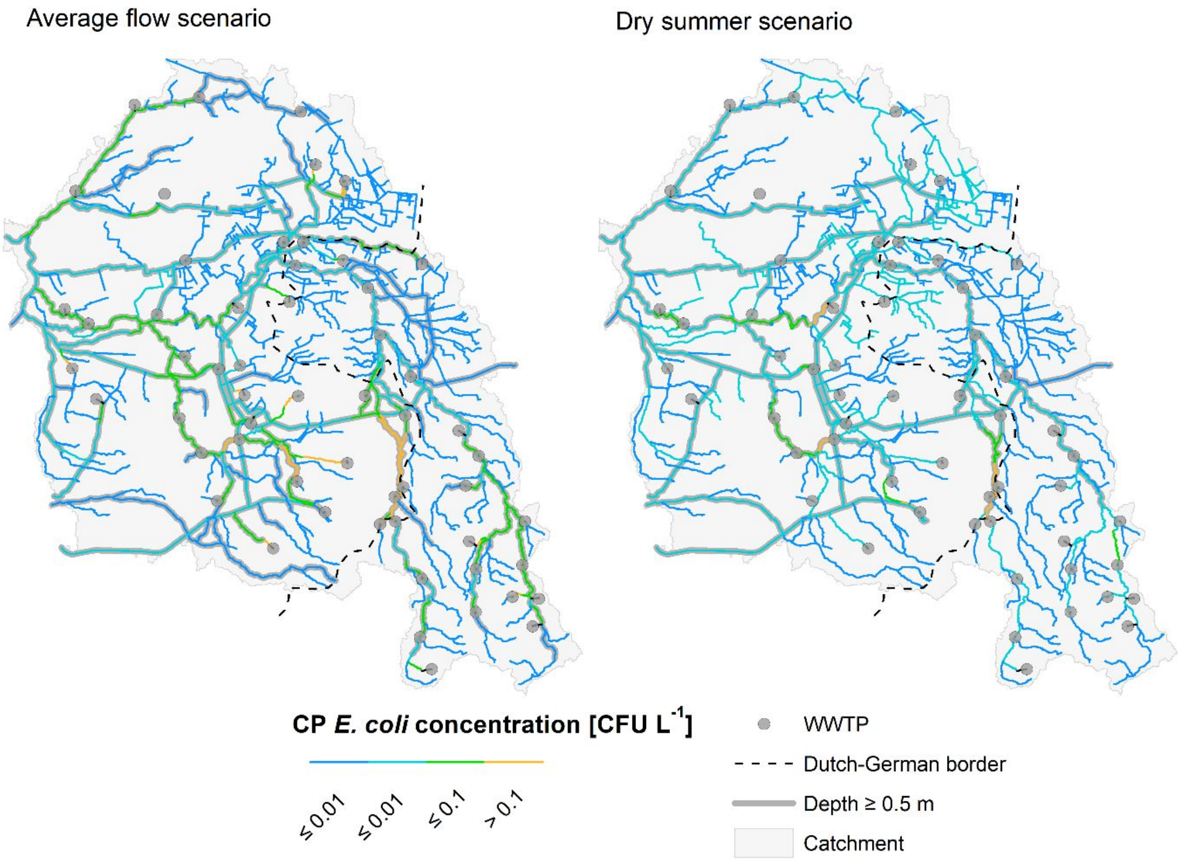

Figure S6. Predicted concentration of CP *E. coli* in the average flow and the dry summer scenario. WWTP = wastewater treatment plant. A depth ≥ indicates potential swimming sites.

Text 5: Impact of modeled processes on *E. coli* concentrations

To assess the impact of WWTP emissions, diffuse emissions, sedimentation and inactivation on predicted *E. coli* concentrations we created scenarios where these processes were excluded. Thus, eight scenarios were created: four on the basis of the average flow scenario and four on the basis of the dry summer scenario. We compared so-derived *E. coli* concentrations ($PEC_{excluded}$) with the respective baseline scenarios ($PEC_{baseline}$), i.e. the average flow scenario and the dry summer scenario, as log difference (*logD*):

$$logD = \log_{10}\left(\frac{PEC_{excluded}}{PEC_{baseline}}\right) = \begin{cases} \log_{10}(PEC_{excluded}) - \log_{10}(PEC_{baseline}) & , PEC_{excluded} > 0 \\ -\infty & , \text{else} \end{cases}$$

A negative *logD* value indicates lower concentrations in the simulation excluding the respective process. On the other hand site, positive values indicate an increase in concentration compared to the baseline scenario. *logD* values are calculated for each river segment. If an exclusion of a process leads to predicted concentrations of 0 CFU L^{-1} *logD* is minus infinity. Results are displayed as cumulative distribution functions in Figure S7 and spatially resolved as maps in Figures S8 – S9. Excluding emission processes leads lower concentrations and excluding loss processes to higher concentrations (Figure S7).

For this analysis, we define that if the exclusion of a process leads to a deviation of less than 0.25 log units in concentration compared to the baseline scenario, the river segment is not sensitive towards the excluded process. Consequently, we call a process "sensitive" towards a river segment, if the deviation is larger than 0.25 log units. Additionally, we call a process "very sensitive" towards a river segment, when it increases or decreases simulated concentrations by more than one order of magnitude.

Due to pumping activities in the catchment, a different proportion of cumulated flow length is affected by wastewater emissions. In the average flow scenario and the dry summer scenario 37% and 53% of cumulated flow length in the Vecht catchment are affected by WWTP emissions. The other river segments are not affected by the exclusion of WWTP emissions (see Figures S8a and S9a). For the average conditions scenario, this process is sensitive for 29% of cumulated flow length. For the dry summer scenario, the

impact of WWTP emissions disappears faster compared to the average flow scenario (see Figures S8a and S9a) so that only 18% of cumulated flow length is sensitive towards WWTP emissions. For both scenarios, less than 10% of cumulated flow lengths are very sensitive towards WWTP emissions.

Trivially, all river sections upstream of any point source are sensitive to diffuse emissions. This accounts for 63% and 47% of cumulated flow length for the average flow scenario and the dry summer scenario, respectively. Additionally, 38% and 70% of cumulated flow lengths downstream of WWTPs are sensitive and 23% and 55% are very sensitive towards diffuse emissions for the average flow scenario and the dry summer scenario, respectively. The impact of diffuse emissions is least sensitive at WWTP discharge sites (see Figures S8 and S9). This is where river segments are most sensitive towards WWTP emissions.

In the model, sedimentation takes place in all river segments. The process depends on the residence time of a segment, calculated by the length of the river segment and the flow velocity as well as on its depth. Both, flow velocity and depth, are generally higher for natural waterbodies in the average flow scenario. Consequently, in the average flow scenario, 72% and 10% of cumulated flow length are sensitive and very sensitive towards sedimentation, whereas in the dry summer scenario 96% and 26% of cumulated flow length are sensitive and very sensitive towards sedimentation (see Figure S7). In canals, the flow velocity is lower compared to natural flowing waterbodies [16]. Therefore, these waterbodies have a comparably longer residence time and are more sensitive towards sedimentation. In the average flow scenario, 96% and 41% of cumulated canal flow length are sensitive and very sensitive towards sedimentation. In the dry summer scenario, flow velocity in some canals can be increased compared to the average flow scenario due to pumping activities. This results in 95% and 32% of cumulated canal flow length being sensitive and very sensitive towards sedimentation.

Just like sedimentation, inactivation is also modeled to occur catchment-wide. In contrast to sedimentation, inactivation is modeled to be independent of the depth of the respective segment.

Generally the inactivation affects concentrations less than sedimentation in both scenarios (Figures S8 and S9). 17% and 19% of cumulated flow length are sensitive towards inactivation, for the average flow scenario and the dry summer scenario, respectively. Less than 1% of cumulated flow length is very sensitive towards inactivation in both scenarios.

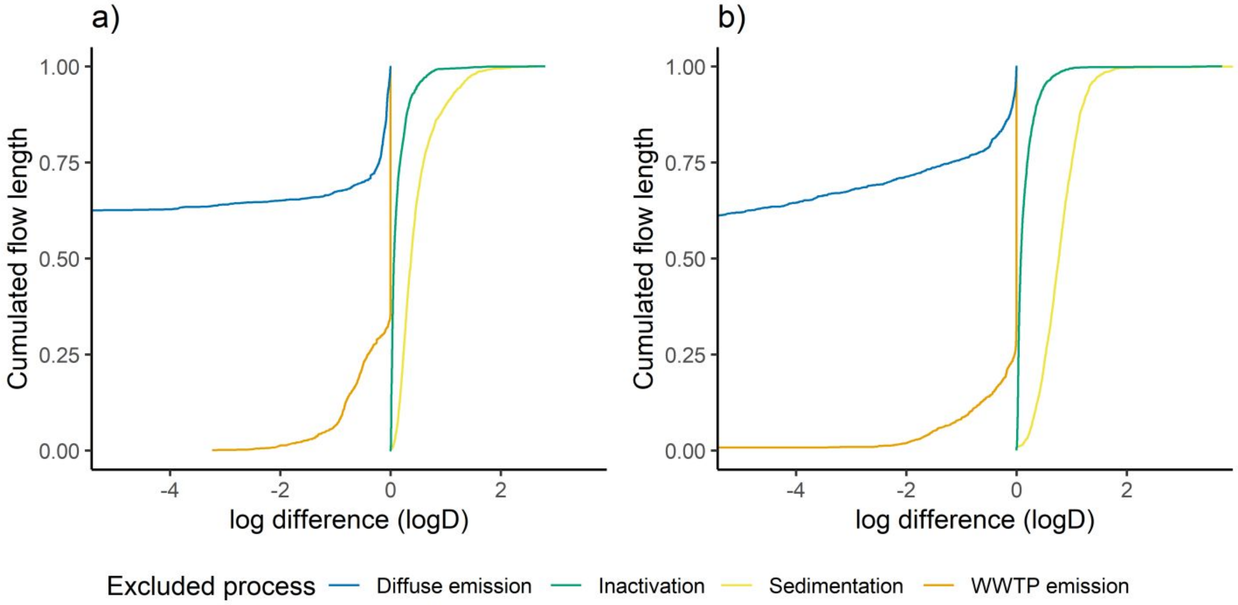

Figure S7. Cumulated flow length of differences between simulations excluding WWTP emissions, diffuse emissions, sedimentation and inactivation and the baseline scenarios, i.e. the average flow scenario (a) and the dry summer scenario (b).

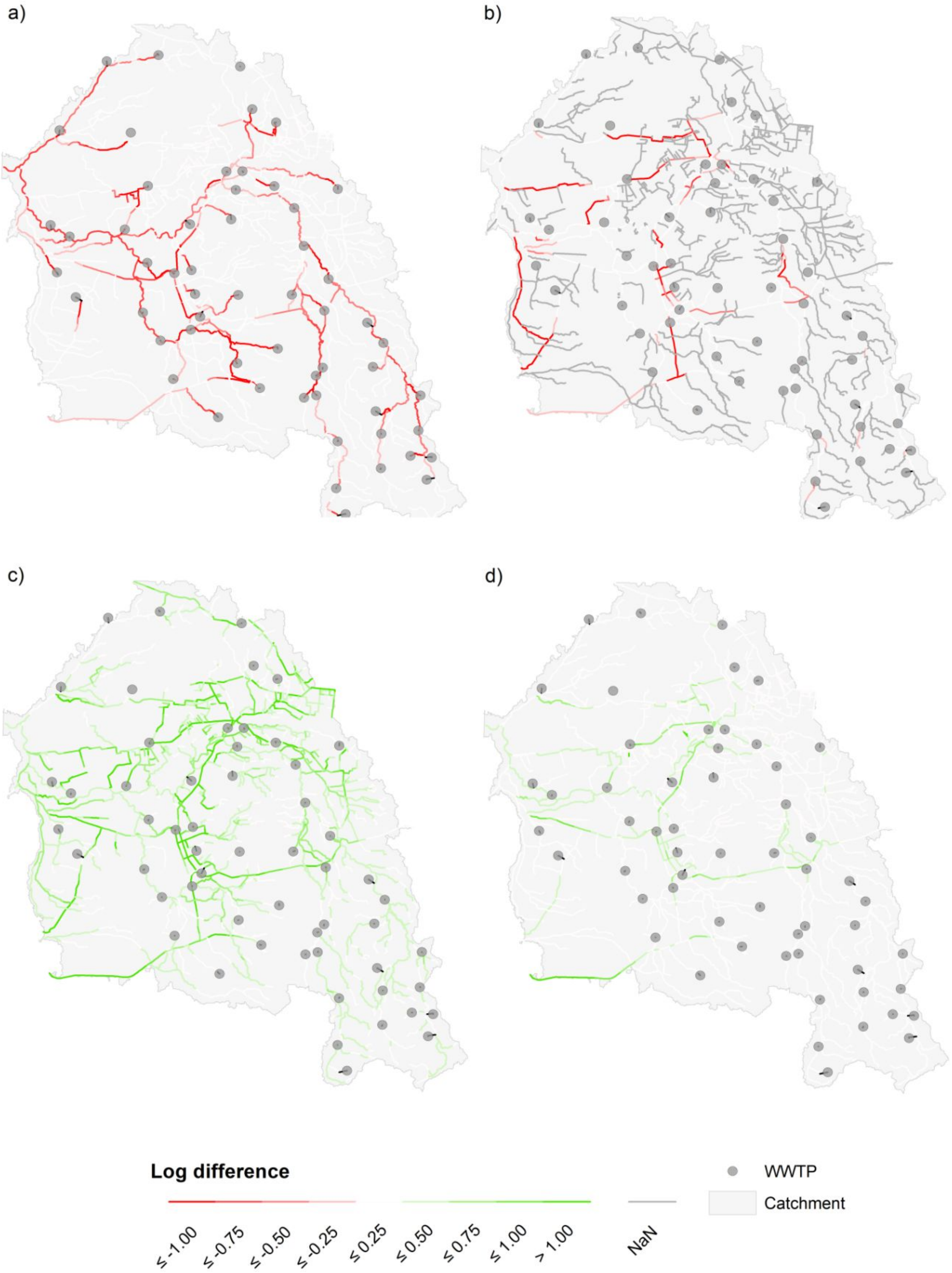

Figure S8. Spatially resolved impact of WWTP emission (a), diffuse emission (b), sedimentation (c), and inactivation (d) for the average flow scenario. Log difference = $\log_{10}(PEC_{excluded}/PEC_{baseline})$. $PEC_{baseline}$ and $PEC_{excluded}$ are predicted environmental concentrations in the scenarios including and excluding a process, respectively. NaN (not a number) values indicate that $PEC_{excluded}$ = 0.

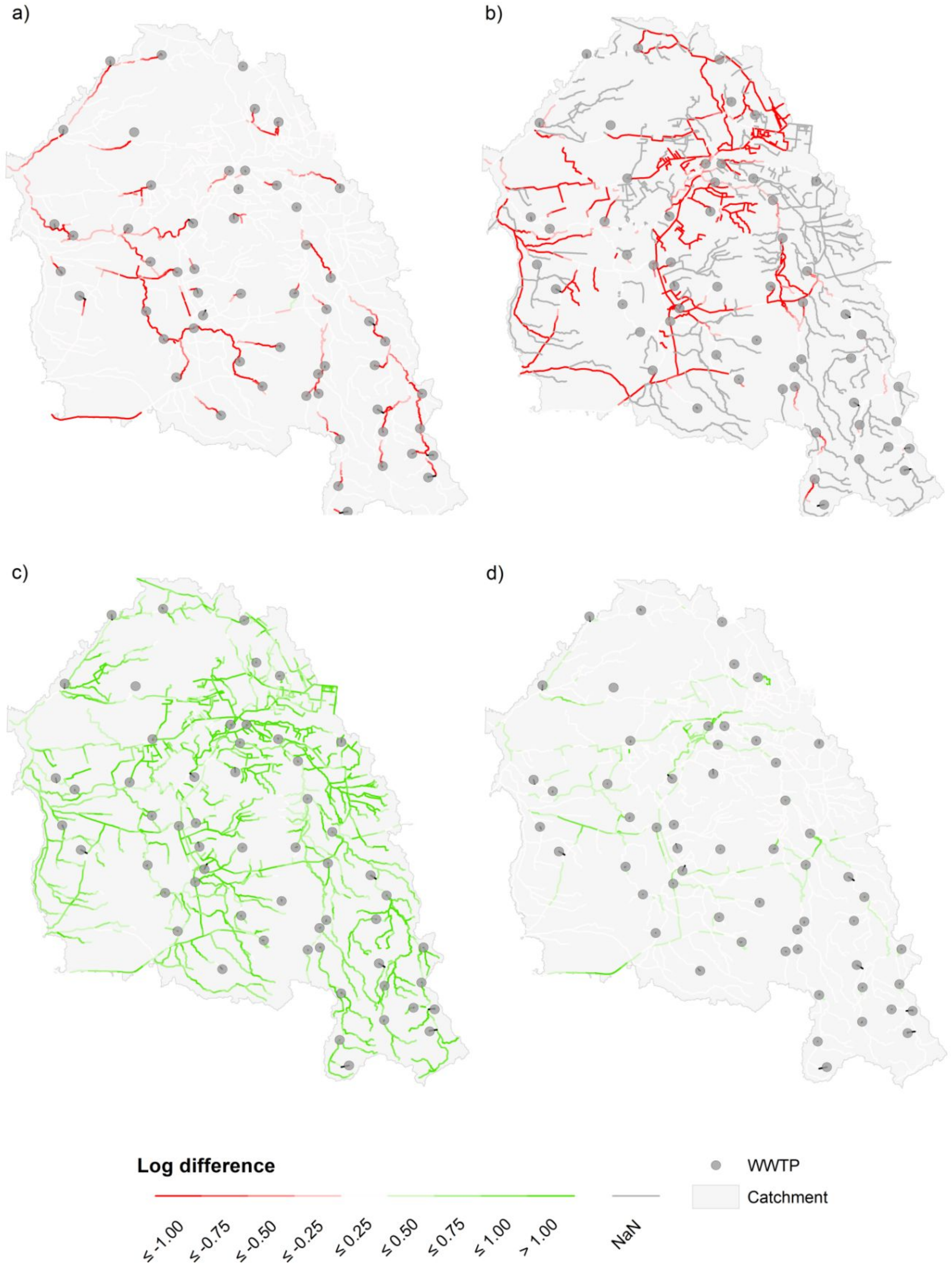

Figure S9. Spatially resolved impact of WWTP emission (a), diffuse emission (b), sedimentation (c), and inactivation (d) for the dry summer scenario. Log difference = $\log_{10}(PEC_{excluded}/PEC_{baseline})$. $PEC_{baseline}$ and $PEC_{excluded}$ are predicted environmental concentrations in the scenarios including and excluding a process, respectively. NaN (not a number) values indicate that $PEC_{excluded} = 0$.

References

1. European Environment Agency, Corine Land Cover (CLC) 2018, Version 2020_20u1. In 2018.
2. NEN, *NEN 6600-2:2009 Water - Sampling - Part 2: Surface water*. The Netherlands Standardization Institute (NEN): Delft, the Netherlands, 2009.
3. ISO, *ISO 8199:2018 Water quality - General requirements and guidance for microbiological examinations by culture*. International Organization for Standardization: Geneva, Switzerland, 2018.
4. ISO, *ISO 16649-2 Microbiology of food and animal feeding stuffs - Horizontal method for the enumeration of β-glucuronidase-positive Escherichia coli - Part 2: Colony-count technique at 44 °C using 5-bromo-4-chloro-3-indolyl β-D-glucuronide*. International Organization for Standardization: Geneva, Switzerland, 2001.
5. Duarte, D. J.; Niebaum, G.; Lämmchen, V.; Heijnsbergen, E.; Oldenkamp, R.; Hernández-Leal, L.; Schmitt, H.; Ragas, A. M. J.; Klasmeier, J., Ecological Risk Assessment of Pharmaceuticals in the Transboundary Vecht River (Germany and The Netherlands). *Environ Toxicol and Chem* **2021**.
6. Jamieson, R.; Joy, D. M.; Lee, H.; Kostaschuk, R.; Gordon, R., Transport and deposition of sediment-associated Escherichia coli in natural streams. *Water Res* **2005,** *39*, (12), 2665-75.
7. Garcia-Armisen, T.; Servais, P., Partitioning and Fate of Particle-AssociatedE. coliin River Waters. *Water Environment Research* **2009,** *81*, (1), 21-28.
8. Characklis, G. W.; Dilts, M. J.; Simmons, O. D., 3rd; Likirdopulos, C. A.; Krometis, L. A.; Sobsey, M. D., Microbial partitioning to settleable particles in stormwater. *Water Res* **2005,** *39*, (9), 1773-82.
9. Fries, J. S.; Characklis, G. W.; Noble, R. T., Attachment of Fecal Indicator Bacteria to Particles in the Neuse River Estuary, N.C. *Journal of Environmental Engineering* **2006,** *132*, (10), 1338-1345.
10. Blaak, H.; Schilperoort, R.; Schmitt, H. *Rol van afvalwater bij verspreiding van antibioticaresistentie. ESBL-producerende Escherichia coli en ampicillineresitstente Enterococcus faecium in oppervlaktewater*; rapport 2018-11; STOWA RIONED: 2018.
11. Pachepsky, Y. A.; Shelton, D. R., Escherichia Coli and Fecal Coliforms in Freshwater and Estuarine Sediments. *Crit Rev Env Sci Tec* **2011,** *41*, (12), 1067-1110.
12. Kim, J.-W.; Pachepsky, Y. A.; Shelton, D. R.; Coppock, C., Effect of streambed bacteria release on E. coli concentrations: Monitoring and modeling with the modified SWAT. *Ecol Model* **2010,** *221*, (12), 1592-1604.
13. Pachepsky, Y.; Stocker, M.; Saldana, M. O.; Shelton, D., Enrichment of stream water with fecal indicator organisms during baseflow periods. *Environ Monit Assess* **2017,** *189*, (2), 51.
14. Park, Y.; Pachepsky, Y.; Hong, E. M.; Shelton, D.; Coppock, C., Release from Streambed to Water Column during Baseflow Periods: A Modeling Study. *J Environ Qual* **2017,** *46*, (1), 219-226.
15. Avery, S. M.; Moore, A.; Hutchison, M. L., Fate of Escherichia coli originating from livestock faeces deposited directly onto pasture. *Letters in Applied Microbiology* **2004,** *38*, (5), 355-359.
16. Lämmchen, V.; Klasmeier, J.; Hernandez-Leal, L.; Berlekamp, J., Spatial Modelling of Micro-pollutants in a Strongly Regulated Cross-border Lowland Catchment. *Environ Process* **2021**.
